# Sex Differences in Cardiovascular Medication Prescription in Primary Care: A Systematic Review and Meta‐Analysis

**DOI:** 10.1161/JAHA.119.014742

**Published:** 2020-06-20

**Authors:** Min Zhao, Mark Woodward, Ilonca Vaartjes, Elizabeth R. C. Millett, Kerstin Klipstein‐Grobusch, Karice Hyun, Cheryl Carcel, Sanne A. E. Peters

**Affiliations:** ^1^ Julius Global Health Julius Centre for Health Sciences and Primary Care Utrecht Medical Centre Utrecht University Utrecht Netherlands; ^2^ The George Institute for Global Health University of Oxford United Kingdom; ^3^ The George Institute for Global Health University of New South Wales Sydney Australia; ^4^ Department of Epidemiology John Hopkins University Baltimore MD; ^5^ Global Geo and Health Data center Utrecht University Utrecht The Netherlands; ^6^ Division of Epidemiology & Biostatistics School of Public Health Faculty of Health Sciences University of the Witwatersrand Johannesburg South Africa; ^7^ Faculty of Medicine and Health Westmead Applied Research Centre University of Sydney Australia; ^8^ Sydney School of Public Health Sydney Medical School University of Sydney New South Wales Australia

**Keywords:** cardiovascular medication, meta‐analysis, primary care, sex differences, systematic review, Epidemiology, Cardiovascular Disease

## Abstract

**Background:**

Sex differences in the management of cardiovascular disease have been reported in secondary care. We conducted a systematic review with meta‐analysis of systematically investigated sex differences in cardiovascular medication prescription among patients at high risk or with established cardiovascular disease in primary care.

**Methods and Results:**

PubMed and Embase were searched between 2000 and 2019 for observational studies reporting on the sex‐specific prevalence of aspirin, statins, and antihypertensive medication prescription, including beta blockers, calcium channel blockers, angiotensin‐converting enzyme inhibitors, and diuretics, in primary care. Random effects meta‐analysis was used to obtain pooled women‐to‐men prevalence ratios for each cardiovascular medication prescription. Metaregression models assessed the impact of age and year on the findings. A total of 43 studies were included, involving 2 264 600 participants (28% women) worldwide. Participants’ mean age ranged from 51 to 76 years. The pooled prevalence of cardiovascular medication prescription for women was 41% for aspirin, 60% for statins, and 68% for any antihypertensive medications. Corresponding rates for men were 56%, 63%, and 69% respectively. The pooled women‐to‐men prevalence ratios were 0.81 (95% CI, 0.72–0.92) for aspirin, 0.90 (95% CI, 0.85–0.95) for statins, and 1.01 (95% CI, 0.95–1.08) for any antihypertensive medications. Women were less likely to be prescribed angiotensin‐converting enzyme inhibitors (0.85; 95% CI, 0.81–0.89) but more likely with diuretics (1.27; 95% CI, 1.17–1.37). Mean age, mean age difference between the sexes, and year of study had no significant impact on findings.

**Conclusions:**

Sex differences in the prescription of cardiovascular medication exist among patients at high risk or with established cardiovascular disease in primary care, with a lower prevalence of aspirin, statins, and angiotensin‐converting enzyme inhibitors prescription in women and a lower prevalence of diuretics prescription in men.

**Nonstandard Abbreviations and Acronyms**
ACEI: angiotensin‐converting enzyme inhibitors
Antihtn: antihypertensive medications
BB: beta blocker
CCB: calcium channel blocker
CHD: coronary heart disease
CVD: cardiovascular disease

**Clinical Perspective**

**What Is New?**

- This systematic review with meta‐analysis shows that there are sex differences in cardiovascular medication prescription among patients at high risk or with established cardiovascular disease in primary care.
- Women were less likely to be prescribed aspirin, statin, or angiotensin‐converting enzyme inhibitor but more likely to have a prescription for diuretics.

**What Are the Clinical Implications?**

- Sex differences in cardiovascular prescription in primary care need to be addressed in order to optimize the use of cardiovascular medication for both women and men.

Cardiovascular disease (CVD) remains the leading cause of death worldwide, accounting for about a third of all deaths in both women and men.^1^ Historically, there has been a misperception that CVD predominantly affects men, which may have resulted in suboptimal management and treatment of CVD in women.^2^, ^3^ Over recent decades, substantial efforts have been made to characterize CVD in women. As a result, important differences between women and men in the presentation, diagnosis, and medical treatment of CVD have been identified.^2^, ^4^

Most studies on sex differences in CVD management have been performed in secondary care.^3^, ^5^, ^6^, ^7^ For example, among all patients receiving statins after hospitalization for myocardial infarction in the United States, women were less likely than men to receive high‐intensity statins, despite guideline recommendations.^6^ Also, a study of coronary heart disease patients recruited from routine outpatient cardiology clinics in 11 countries across Europe, Asia, and the Middle East showed that women were less likely than men to reach all treatment targets set by clinical guidelines.^3^ Whether similar sex differences exist in primary care has not been systematically evaluated. Considering that both patients at high risk and with established CVD attended clinics in primary care to monitor their current CVD treatment, primary care visits are a key stage at which any sex inequities in treatment could and should be investigated. Comprehensive evidence on current sex differences in cardiovascular medication prescription in primary care would help to obtain a better understanding of the utilization of evidence‐based medical treatment for both sexes and encourage all health professionals to strive for sex equity in providing CVD management to their patients.

In this study, we conducted a systematic review and meta‐analysis to determine the prevalence of common cardiovascular medication prescription in women and men in primary care and to evaluate whether prescriptions for guideline‐recommended cardiovascular medications differ between the sexes.

## Methods

The authors declare that all supporting data are available within the article and its online supplementary files.

### Search Strategy

A systematic search of observational studies was performed in PubMed/MEDLINE and Embase for studies published between 2000 and 2019 using combined text word subject heading terms (Table S1). The reference lists of all related articles were screened for any other potentially relevant studies.

### Study Selection and Data Extraction

All observational studies that reported the sex‐specific prevalence of prescriptions of cardiovascular medications (aspirin, statins, and any antihypertensive medication including beta blockers, calcium channel blockers [CCBs], angiotensin‐converting enzyme inhibitors [ACE inhibitors], and diuretics) for patients at high risk or with established CVD (coronary heart disease, stroke, heart failure, and atrial fibrillation) in primary care were included. Studies were excluded if they (1) were published in a language other than English; (2) presented an unrelated study population, outcome, or were not performed in primary care; (3) included <1000 patients; (4) reported cardiovascular medication prescription only for 1 sex; and (5) assessed cardiovascular medication not by prescription (such as self‐report or pharmacy dispensing).

Duplicate records were removed before title and abstract screening. When there were multiple reports from the same study, the report involving the highest number of cases or most explicit participants characteristics and outcome measures was included. Four independent reviewers (M.Z., E.R.C.M., C.C., and K.H.) screened the papers by title and abstract against the inclusion and exclusion criteria. Any disagreement between reviewers was discussed and the full text was reviewed, if necessary. A similar process took place in reviewing the full text of selected papers. A tailor‐made data extraction form was used to collect information on study and participant characteristics and sex‐specific prevalence of prescriptions of cardiovascular medication (Table S2).

### Quality Assessment

Study quality was assessed using the modified Newcastle‐Ottawa scale for observational studies. This scale consists of 6 items that assess the quality of participant selection, comparability, and outcome adjudication (Tables S3 and S4).^8^

### Outcomes

The primary outcome was the women‐to‐men prescription prevalence ratio with 95% CI for each cardiovascular medication. The secondary outcomes were the sex‐specific prescription rates of each cardiovascular medication.

### Statistical Analysis

In general, the included studies reported unadjusted numbers, rates, or percentages of women and men with cardiovascular medication prescriptions. If a measure of variability was not reported, these were estimated from the rate and the sample size. The women‐to‐men prevalence ratios with 95% CI were pooled across studies using random‐effects meta‐analyses with inverse‐variance weighting for each medication.^9^ In sensitivity analysis, we pooled the results from studies that had adjusted for age. As different studies reported on different antihypertensive medications, we also restricted the analyses on individual antihypertensive medications to studies that reported on each of the 4 antihypertensive medications. Metaregression analyses were performed to assess the impact of mean age and age difference (women minus men) on our findings. We further investigated whether there was a trend in sex differences in cardiovascular medication prescription over time. In subgroup analysis, we assessed whether the findings differed by CVD status (high risk only, prevalent CVD, and high risk and prevalent CVD combined). *P*<0.05 were considered statistically significant. Statistical analyses were performed by using the “metafor” package in R version 3.2.2.

## Results

### Study Characteristics

**Figure 1:**
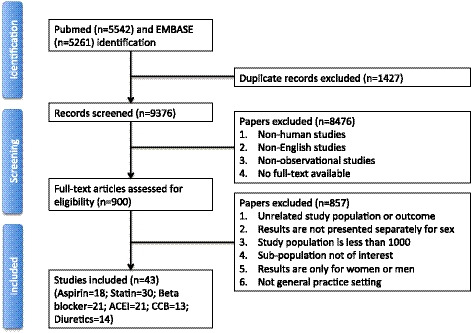
Flowchart of records screened and included in the systematic review. ACEI indicates angiotensin‐converting enzyme inhibitor; and CCB, calcium channel blocker.

Of the 10 803 studies identified through the systematic search, 900 studies were reviewed in full text (Figure 1). Of these, 43 studies were included, including a total of 2 264 600 participants, of whom 630 111 (28%) were women. The mean age ranged from 51 to 76 years (where reported). Table shows the key characteristics of the included studies. Of the 43 studies, 18 included information on aspirin,^10^, ^11^, ^12^, ^13^, ^14^, ^15^, ^16^, ^17^, ^18^, ^19^, ^20^, ^21^, ^22^, ^23^, ^24^, ^25^, ^26^, ^27^ 30 on statins,^*^ 14 on any antihypertensive medications,^†^ 21 on beta blockers,^‡^ 13 on CCBs,^§^ 21 on ACE inhibitors,^||^ and 14 on diuretics.^¶^ Eight out of 43 studies reported cardiovascular medication prescription for high‐risk patients,^17^, ^32^, ^38^, ^47^, ^48^, ^49^, ^52^, ^53^ 24 for patients with established CVD,^#^ and 11 for both high‐risk and CVD patients.^**^

**Table 1:** Key Characteristics of Selected Studies

Study	Year	Country	Prevention Type	Sample Size	Women	Men	Age, y	Cardiovascular Medications						
								Aspirin	Statin	Antihtn	BB	CCB	ACEI	Diuretics
Al‐Lawati et al^10^	2007	Oman	Mixed	2551	1352	1199	54	X		X	X	X	X	X
Alberts et al^28^	2004	Multiple	Secondary	55 499	18 315	37 184	69		X					
Brady et al^11^	1998	UK	Secondary	24 431	9898	14 533	67	X	X		X	X	X	X
Brady et al^20^	2002	UK	Secondary	12 045	4457	7588	67	X	X		X		X	X
Bull et al^31^	2003^a^	UK	Secondary	13 929	5827	8102	>40		X					
Carlsson et al^21^	2013^a^	Sweden	Secondary	7408	3330	4078	76	X	X		X	X		X
Carroll et al^22^	2001	UK	Secondary	6778	2787	3991	NA	X	X		X			
Catalán‐Ramos et al^32^	2009	Spain	Primary	696 073	358 218	337 855	51		X		X	X	X	X
Crilly et al^23^	2001	UK	Secondary	1162	552	610	69	X	X		X			
Dodhia et al^33^	2013	UK	Secondary	6711	2828	4564	70		X					
Dreyer et al^34^	2007	Australia	Secondary	2005	721	1284	70		X		X	X	X	
Driscoll et al^24^	2007	Australia	Secondary	12 509	5267	7242	73	X		X				
Emberson et al^25^	2001	UK	Mixed	8538	4286	4252	NA	X	X	X	X		X	
Forster et al^35^	2013	UK	Secondary	23 811	4502	4252	NA		X	X				
Greving et al^49^	2000	NL	Primary	7550	4774	2776	63				X	X	X	X
Gulliford et al^36^	2010^a^	UK	Secondary	7065	3816	3249	73		X	X				
Hawkins et al^50^	2007	UK	Secondary	13 330	6803	6527	68				X		X	
Hendrix et al^26^	2005^a^	US	Mixed	72 508	29 208	43 300	NA	X	X		X	X	X	X
Hippisley‐Cox et al^27^	2001^a^	UK	Mixed	5891	2783	3108	NA	X			X			
Hyun et al^37^	2012	Australia	Mixed	13 294	6202	7092	61		X	X				
Journath et al^38^	2005	Sweden	Primary	6537	3410	3127	66		X	X	X	X	X	X
Lahoz et al^12^	2008^a^	Spain	Secondary	8817	2319	6498	65	X	X		X	X	X	X
Law et al^44^	2010	Canada	Primary	390	128	262	58		X					
Lawlor et al^29^	2000	UK	Secondary	1314	483	831	NA		X					
Lee et al^19^	2018	Australia	Secondary	130 926	61 142	69 784	67	X	X	X	X		X	
Macchia et al^13^	2012^a^	Italy	Secondary	21 423	6928	14 495	NA	X	X		X		X	
Majeed et al^39^	1996	UK	Secondary	63 259	34 545	28 714	NA		X					
Majeed et al^14^	2002	UK	Secondary	2129	1224	905	NA	X					X	X
Murphy et al^51^	2004^a^	UK	Secondary	2186	1213	973	NA				X		X	X
Nanna et al^46^	2015	US	Mixed	5693	2460	3233	68		X					
Nilsson et al^40^	2004^a^	Sweden	Mixed	9375	4293	5082	65		X				X	
Nilsson et al^52^	2007^a^	Sweden	Primary	1135	714	421	52					X	X	X
Owen et al^47^	2009^a^	Australia	Primary	12 499	5896	6603	63			X	X	X	X	X
Paulsen et al^48^	2011^a^	Denmark	Primary	5413	3305	2108	66			X	X	X	X	X
Qato et al^15^	2011	US	Mixed	4136	2233	1903	52	X	X					
Saposnik et al^30^	2004	Canada	Secondary	1094	415	679	67		X	X				
Sheppard et al^41^	2009	UK	Mixed	4699	1937	2762	54		X					
Svilaas et al^16^	2000^a^	Norway	Secondary	2060	707	1353	69	X						
Tabenkin et al^17^	2004	US	Primary	407	210	197	53	X		X			X	
Turnbull et al^42^	2008	Australia	Mixed	3664	1834	1830	68		X	X				
Virani et al^43^	2011	US	Secondary	972 532	13 371	959 161	71		X					
Weler et al^18^	2003	US	Mixed	3849	1953	1896	65	X		X				
Wandell et al^45^	2007	Sweden	Secondary	7975	3465	4510	NA		X		X	X	X	X

ACEI indicates angiotensin converting enzyme inhibitor; Antihtn, any anti‐hypertensive medication; BB, beta blocker; CCB, calcium channel blocker; EU, Europe; NL, The Netherlands; UK, United Kingdom; and US, United States.

a Year: study performed year. Studies with asterisk indicate publication year.

### Sex Differences in Prevalence of Cardiovascular Medication Prescription

**Figure 2:**
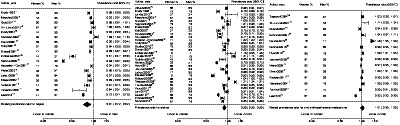
Women‐to‐men prevalence ratio of aspirin, statins, and any antihypertensive medications prescription. For each study, the square is centered on the women‐to‐men prevalence ratio and the horizontal lines show the associated 95% CI. The diamond indicates the pooled summary and its 95% CI.

In women, the pooled prevalence of cardiovascular medication prescription was 41% for aspirin, 60% for statins, and 68% for overall antihypertensive medications. The corresponding rates for men were 56%, 63%, and 69%, respectively. The pooled women‐to‐men prevalence ratios were 0.81 (95% CI, 0.72–0.92) for aspirin, 0.90 (95% CI, 0.85–0.95) for statins, and 1.01 (95% CI, 0.95–1.08) for any antihypertensive medications (Figure 2).

**Figure 3:**
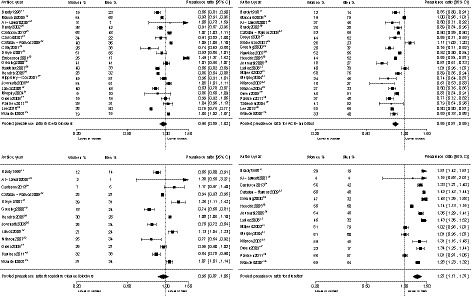
Women‐to‐men prevalence ratio of individual antihypertensive medication prescription. For each study, the square is centered on the women‐to‐men prevalence ratio and the horizontal lines show the associated 95% CI. The diamond indicates the pooled summary and its 95% CI.

Figure 3 shows the women‐to‐men prevalence ratios of individual antihypertensive medication prescription. Women were less likely to be prescribed with ACE inhibitors (women‐to‐men prevalence ratio: 0.85; 95% CI, 0.81–0.89) whereas the prevalence of diuretics prescription was higher than in men (women‐to‐men prevalence ratio: 1.27; 95% CI, 1.17–1.37). There were no significant sex differences in the prescription of beta blockers and CCBs. Findings were similar in analyses restricted to studies that reported on all 4 individual antihypertensive medications (Figure S1). Findings were similar in age‐adjusted analyses, available for 31 studies (Tables S5 through S10).

### Impact of Age on the Sex Differences in Prevalence of Cardiovascular Medication

Among the 31 studies that reported a sex‐combined mean age of the study population, there was no evidence that the women‐to‐men prevalence ratio varied systematically according to the mean age (Figure S2; *P* values: 0.57 for aspirin; 0.24 for beta blockers; 0.27 for CCBs; 0.41 for ACE inhibitors; 0.85 for diuretics). The only exception was that in studies with older patients, women were less likely than men to be prescribed statins whereas women had a higher prevalence of statin prescription compared with men in studies including younger patients (*P*=0.003).

Among the 17 studies that reported sex‐specific mean ages, there was no evidence that the prevalence ratio varied systematically according to the women to men age difference (Figure S3; *P* values: 0.34 for aspirin; 0.21 for statins; 0.93 for beta blockers; 0.91 for CCBs; 0.89 for ACE inhibitors). The exception was the higher prevalence of diuretics prescription in women increased as the difference between the mean age of women and the mean age of men increased (*P*=0.006).

### Sex Differences in the Prevalence of Cardiovascular Medication Prescription Over Time

The sex differences in prevalence ratio of prescription did not significantly change over time for aspirin (*P*=0.92), any antihypertensive medications (*P*=0.99), beta blockers (*P*=0.43), CCBs (*P*=0.44), ACE inhibitors (*P*=0.39), and diuretics (*P*=0.58) (Figure S4). However, the pattern and magnitude of the sex differences in statin prescription changed over time, with an increased women‐to‐men prevalence ratio (*P*=0.003).

### Sex Differences in Cardiovascular Medication Prescription by CVD Status

Among patients with established CVD, women were less likely to be prescribed with aspirin (0.89, 95% CI, 0.84–0.94), statins (0.85; 95% CI, 0.80–0.90), beta blockers (0.90, 95% CI, 0.85–0.96), and ACE inhibitors (0.88, 95% CI, 0.84–0.93) (Figure S5, Table S11). In contrast, women with established CVD were more likely to be prescribed with diuretics than their male counterparts (1.25; 95% CI, 1.09–1.43). Similar pooled estimates, but with wider CIs, were found when studies included only high‐risk participants, or when studies included both participants at high risk of and with established CVD. Time trends in the women‐to‐men prevalence ratio in medication prescription did not differ materially by CVD status (Figures S6 through S11). However, the women‐to‐men ratio of statin prescription increased over time in studies among high‐risk patients but not in studies including patients with established CVD or in studies including both high‐risk and CVD patients (*P* for interaction=0.002).

## Discussion

In this systematic review and meta‐analysis of 43 studies including over 2 million participants, we found that there were sex differences in cardiovascular medication prescription among patients at high risk or with established CVD in primary care. Compared with men, women were less likely to have a prescription for aspirin, statins, or ACE inhibitors but more likely to have a prescription for diuretics. Sex differences did not vary materially by age, but there was some evidence to suggest that the magnitude of sex differences in statin prescription increased over time.

Previous studies in secondary care have demonstrated that women are generally less likely than men to have a prescription of guideline‐recommended cardiovascular medications after a cardiac event.^2^, ^3^, ^5^, ^54^ SUrvey of Risk Factors, a clinical audit with over 10 000 patients from 11 countries, indicated that women had a lower prevalence of cardiovascular medication use than men and were less likely to reach treatment targets.^3^ Similarly, a study of 36 000 patients with established coronary heart disease in the United States, showed that women were less likely than men to be prescribed with aspirin, ACE inhibitors, or statins at both acute and hospital discharge of coronary heart disease.^55^ A study in the United Kingdom showed that prescription rates for cardiovascular medications were about 10% lower among women than men <55 years for acute myocardial infarction.^56^ Furthermore, a Dutch population‐based analysis also found persistent sex differences in the use of lipid‐lowering medications for secondary prevention of CVD, particularly in younger patients.^5^ We did not observe that sex disparities differed between age groups, but we noticed that the sex differences in statin prescription persisted and was even larger in the more recent studies. A recent study in the United States confirmed that women were 9% less likely than men to receive high‐intensity statins, as opposed to other types of statin.^57^ The present study further expands these findings by showing that sex differences in medication prescription also exist among patients at high cardiovascular risk or with established CVD in a primary care setting. We also demonstrated that women were more likely to be on diuretics but less likely to be on ACE inhibitors, which is in line with other studies.^56^, ^58^, ^59^ Sex differences in progression and presentation of CVD and comorbidities, the efficiency of treatment, and/or adverse drug effects may lead to different requirements on antihypertensive regimens.^59^, ^60^ The reasons for the contrasting sex differences within antihypertensive medication classes require further study.

There are several other possible explanations for the lower prescription rates of some cardiovascular medications in women than men. First, the incidence of CVD in women is, typically, about a third that of men in middle age and occurs in men about a decade earlier than women, which might have led to the misperception that CVD is less common in women and does not have to be prevented as intensively as in men.^4^, ^34^, ^61^ Additionally, women may have a lower awareness of the severity of their disease and of appropriate CVD treatment and receive less support from healthy providers, compared with men, resulting in lower health consciousness and less frequent use of healthcare services.^5^, ^62^, ^63^, ^64^

Although beyond the scope of the current investigation, studies have reported a considerable delay in receiving appropriate medical treatment to reduce the risk of incident or recurrent cardiac event in women.^2^, ^23^, ^62^, ^63^ Also, women may have less belief than men in the safety and effectiveness of cardiovascular medications and have been reported to have a greater risk of suffering adverse drug reactions, which may lead to a higher discontinuation rate of cardiovascular medications.^60^, ^65^, ^66^, ^67^ Indeed, studies have shown that women have a poorer adherence to cardiovascular medication than men in primary care.^68^, ^69^ These factors would be expected to produce a wider disparity between the usage of cardiovascular medications than our study of prescriptions suggests.

We conducted a large‐scale systematic review with meta‐analyses on sex differences in cardiovascular medication prescription among patients at high risk or with established CVD in a primary care setting. We included all major cardiovascular medications and found that our results were generally robust across patient characteristics. Limitations of this study are inherent to its design and include the differences across studies in design, population, and end point definition.^9^ We had no information on potential combinations of cardiovascular medications prescribed, nor were we able to adjust our findings to potentially important comorbidities or other characteristics. However, some cardiovascular medications target the same risk factor and the lower use of ACE inhibitors among women, relative to men, could be explained by women's higher use of diuretics. Also, we considered sex differences only in medication prescription and were not able to determine whether those differences, where found, resulted in different levels of risk factor control and event rates. Furthermore, patients with established CVD seen in primary care may also receive treatment from secondary care. Also, it is not clear whether general practitioners or cardiologists would be the main source of prescriptions in any individual case. Finally, as the studies included in this review were conducted in mostly high‐income countries, the generalizability of our findings to low‐ and middle‐income countries needs to be assessed.

In conclusion, this meta‐analysis, summarizing all recent literature, shows that sex differences in cardiovascular medication prescription persist in primary care. Future research is needed to determine the underlying causes of observed sex differences and to develop tailored strategies to optimize the use of evidence‐based cardiovascular medication for both women and men.

## Sources of Funding

Zhao is supported by a grant from the Netherlands Organization for Scientific Research (NWO; grant number: 0.22.005.021). Woodward is supported by National Health and Medical Research Council (NHMRC) Australia project grant 632507 and Fellowship APP1080206. Vaartjes is supported by a grant from the Dutch Heart Foundation (grant DHF project “Facts and Figures”). Hyun is supported by National Heart Foundation Australia Postdoctoral Fellowship (102138). Peters is supported by a UK Medical Research Council Skills Development Fellowship (MR/P014550/1).

## Disclosures

Woodward is a consultant to Amgen and Kirin. The remaining authors have no disclosures to report.

## Supporting information


**Tables S1–S11**
**Figures S1–S11**
**References 10–52**

## Notes

1 References 11, 12, 13, 15, 19, 20, 21, 22, 23, 25, 26, 28, 29, 30, 31, 32, 33, 34, 35, 36, 37, 38, 39, 40, 41, 42, 43, 44, 45, 46.

2 References 10, 17, 18, 19, 24, 25, 30, 35, 36, 37, 38, 42, 47, 48.

3 References 10, 11, 12, 13, 19, 20, 21, 22, 23, 25, 26, 27, 32, 34, 38, 45, 47, 48, 49, 50, 51.

4 References 10, 11, 12, 21, 26, 32, 34, 38, 45, 47, 48, 49, 52.

5 References 10, 11, 12, 13, 14, 17, 19, 20, 25, 26, 32, 34, 38, 40, 45, 47, 48, 49, 50, 51, 52.

6 References 10, 11, 12, 14, 21, 26, 32, 38, 45, 47, 48, 49, 51, 52.

7 References 11, 12, 13, 14, 16, 19, 20, 21, 22, 23, 24, 28, 29, 30, 31, 33, 34, 35, 36, 39, 43, 45, 50, 51.

8 References 10, 15, 18, 25, 26, 27, 37, 40, 41, 42, 46.

## References

[1] GBD 2013 Mortality and Causes of Death Collaborators . Global, regional, and national age‐sex specific all‐cause and cause‐specific mortality for 240 causes of death, 1990–2013: a systematic analysis for the Global Burden of Disease Study 2013. Lancet. 2015;117–171. 10.1016/S0140-6736(14)61682-2 PMC4340604 25530442

[2] Cho L , Hoogwerf B , Huang J , Brennan DM , Hazen SL . Gender differences in utilization of effective cardiovascular secondary prevention: a Cleveland Clinic prevention database study. J Womens Health (Larchmt). 2008;515–521. 18345999 10.1089/jwh.2007.0443 PMC2836534

[3] Zhao M , Vaartjes I , Graham I , Grobbee D , Spiering W , Klipstein‐Grobusch K , Woodward M , Peters SAE . Sex differences in risk factor management of coronary heart disease across three regions. Heart. 2017;1587–1594. 28931567 10.1136/heartjnl-2017-311429 PMC5739833

[4] Woodward M . Cardiovascular disease and the female disadvantage. Int J Environ Res Public Health. 2019;1165. 10.3390/ijerph16071165 PMC6479531 30939754

[5] Koopman C , Vaartjes I , Heintjes EM , Spiering W , van Dis I , Herings RM , Bots ML . Persisting gender differences and attenuating age differences in cardiovascular drug use for prevention and treatment of coronary heart disease, 1998–2010. Eur Heart J. 2013;3198–3205. 24046432 10.1093/eurheartj/eht368

[6] Dallongevillle J , De Bacquer D , Heidrich J , De Backer G , Prugger C , Kotseva K , Montaye M , Amouyel P ; EUROASPIRE Study Group . Gender differences in the implementation of cardiovascular prevention measures after an acute coronary event. Heart. 2010;1744–1749. 20956490 10.1136/hrt.2010.196170

[7] De Smedt D , De Bacquer D , De Sutter J , Dallongeville J , Gevaert S , De Backer G , Bruthans J , Kotseva K , Reiner Z , Tokgozoglu L , et al. The gender gap in risk factor control : effects of age and education on the control of cardiovascular risk factors in male and female coronary patients. The EUROASPIRE IV study by the European Society of Cardiology. Int J Cardiol. 2016;284–290. 10.1016/j.ijcard.2016.02.015 26913370

[8] Wells G , Shea B , O'Connell D , Peterson J , Welch V , Losos M , Tugwell P . The Newcastle‐Ottawa Scale (NOS) for assessing the quality of nonrandomised studies in meta‐analyses. Available at: http://www.ohri.ca/progr​ams/clini​cal_epide​miolo​gy/oxford.asp. Accessed May 5, 2018.

[9] Woodward M . Rationale and tutorial for analysing and reporting sex differences in cardiovascular associations. Heart. 2019;​1701–1708. 10.1136/heartjnl-2019-315299 PMC6855792 31371439

[10] Al‐lawati JA , Barakat MN , Al‐zakwani I , Elsayed MK , Al‐Maskari M , M Al‐Lawati N , Mohammed AJ . Control of risk factors for cardiovascular disease among adults with previously diagnosed type 2 diabetes mellitus: a descriptive study from a Middle Eastern Arab population. Open Cardiovasc Med J. 2012;133–140. 23166566 10.2174/1874192401206010133 PMC3496907

[11] Brady AJB , Oliver MA , Pittard JB . Secondary prevention in 24 431 patients with coronary heart disease: survey in primary care. BMJ. 2001;1463. 11408303 10.1136/bmj.322.7300.1463 PMC32309

[12] Lahoz C , Mantilla T , Taboada M , Soler B , Tranche S , Lopez‐Rodriguez I , Monteiro B , Martin‐Jadraque R , Sanchez‐Zamorano MA , Mostaza JM . Gender differences in evidence‐based pharmacological therapy for patients with stable coronary heart disease. Int J Cardiol. 2009;336–340. 10.1016/j.ijcard.2007.12.115 18486250

[13] Macchia A , Romero M , Ettorre AD , Mariani J , Tognoni G . Temporal trends of the gaps in post‐myocardial infarction secondary prevention strategies of co‐morbid and elderly populations vs. younger counterparts : an analysis of three successive cohorts between 2003 and 2008. Eur Heart J. 2017;515–522. 10.1093/eurheartj/ehr410 22096090

[14] Majeed A , Williams J , De Lusignan S , Chan T . Management of heart failure in primary care after implementation of the National Service Framework for Coronary Heart Disease: a cross‐sectional study. Public Health. 2005;105–111. 10.1016/j.puhe.2004.06.006 15694957

[15] Qato DM , Lee TA , Durazo‐arvizu R , Wu D , Wilder J , Reina SA , Cai J , Gonzalez F II , Talavera GA , Ostfeld RJ , Daviglus ML . Statin and aspirin use among Hispanic and Latino adults at high study/study of Latinos. J Am Heart Assoc. 2016;e002905 DOI: 10.1161/JAHA.115.002905. 27030340 PMC4859281

[16] Svilaas A , Thoresen M , Kristoffersen JE , Hjartaaker J , Westheim A . How well are patients with atherosclerotic disease treated? Secondary prevention in primary care. Scand J Prim Health Care. 2000;232–236. 11205092 10.1080/028134300448805

[17] Tabenkin H , Eaton CB , Roberts MB , Parker DR , McMurray JH , Borkan J . Differences in cardiovascular disease risk factor management in primary care by sex of physician and patient. Ann Fam Med. 2010;25–32. 20065275 10.1370/afm.1071 PMC2807384

[18] Weler DJ , Nathan DM , Grant RW , Meigs JB , Nathan DM , Cagliero E . Sex disparities in treatment of cardiac risk factors in patients with type 2. Diabetes Care. 2005;514–520. 15735180 10.2337/diacare.28.3.514

[19] Lee C , Mnatzaganian G , Woodward M , Chow CK , Sitas F , Robinson S , Huxley RR . Sex disparities in the management of coronary heart disease in general practices in Australia. Heart. 2019;1898–1904. 31337667 10.1136/heartjnl-2019-315134

[20] Brady AJB , Pittard JB , Grace JF , Robinson PJ . Clinical assessment alone will not benefit patients with coronary heart disease: failure to achieve cholesterol targets in 12,045 patients‐the Healthwise II study. Int J Clin Pract. 2005;342–345. 15857334 10.1111/j.1742-1241.2005.00365.x

[21] Carlsson AC , Sundquist K , Johansson S , Sundquist J . Differences and time trends in drug treatment of atrial fibrillation in men and women and doctors’ adherence to warfarin therapy recommendations. Eur J Clin Pharmacol. 2013;245–253. 22684091 10.1007/s00228-012-1322-6

[22] Carroll K , Majeed A , Firth C , Gray J . Prevalence and management of coronary heart disease in primary care: population‐based cross‐sectional study using a disease register. J Public Health Med. 2017;29–35. 10.1093/pubmed/fdg007 12669915

[23] Crilly M , Bundred P , Hu X , Leckey L , Johnstone F . Gender differences in the clinical management of patients with angina pectoris: a cross‐sectional survey in primary care. BMC Health Serv Res. 2007;142. 17784961 10.1186/1472-6963-7-142 PMC2034556

[24] Driscoll A , Beauchamp A , Lyubomirsky G , Demos L , McNeil J , Tonkin A . Suboptimal management of cardiovascular risk factors in coronary heart disease patients in primary care occurs particularly in women. Intern Med J. 2011;730–736. 21627740 10.1111/j.1445-5994.2011.02534.x

[25] Emberson JR , Whincup PH , Lawlor DA , Montaner D , Ebrahim S . Coronary heart disease prevention in clinical practice: are patients with diabetes special? Evidence from two studies of older men and women. Heart. 2005;451–456. 15772196 10.1136/hrt.2004.035832 PMC1768806

[26] Hendrix KH , Mayhan S , Lackland DT , Egan BM . Prevalence, treatment, and control of chest pain syndromes and associated risk factors in hypertensive patients. Am J Hypertens. 2017;1026–1032. 10.1016/j.amjhyper.2005.02.016 16109315

[27] Hippisley‐cox J , Pringle M , Crown N , Meal A , Wynn A . Sex inequalities in ischaemic heart disease in general practice: cross sectional survey. BMJ. 2001;1–5. 10.1136/bmj.322.7290.832 PMC30561 11290638

[28] Alberts MJ , Bhatt DL , Mas J , Ohman EM , Hirsch AT , Rother J , Salette G , Goto S , Smith SC Jr , Liau CS , et al.; REduction of Atherothrombosis for Continued Health Registry Investigators . Three‐year follow‐up and event rates in the international REduction of Atherothrombosis for Continued Health Registry. Eur Heart J. 2009;2318–2326. 19720633 10.1093/eurheartj/ehp355 PMC2755116

[29] Lawlor DA , Whincup P , Emberson JR , Rees K , Walker M , Ebrahim S . The challenge of secondary prevention for coronary heart disease in older patients: findings from the British Women's Heart and Health Study and the British Regional Heart Study. Fam Pract. 2004;582–586. 15367482 10.1093/fampra/cmh516

[30] Saposnik G , Goodman SG , Leiter LA , Yan RT , Fitchett DH , Bayer NH , Casanova A , Yan AT ; Vascular Protection; Guidelines‐Oriented Approach to Lipid‐Lowering Registries Investigators; Stroke Outcome Research Canada Working Group . Applying the evidence do patients with stroke, coronary artery disease, or both achieve similar treatment goals? Stroke. 2009;1417–1424. 19213947 10.1161/STROKEAHA.108.533018

[31] Bull N , Williams J , Nicholls P , Lawrenson R . Increased statin prescribing in patients with diabetes after the introduction of the NSF for Coronary Heart Disease. Pract Diabetes Int. 2003;313–317.

[32] Catalán‐Ramos A , Verdú JM , Grau M , Iglesias‐Rodal M , del Val Garcia JL , Consola A , Comin E ; GPC‐ICS Group . Population prevalence and control of cardiovascular risk factors: what electronic medical records tell us. Aten Primaria. 2014;15–24. 10.1016/j.aprim.2013.06.004 PMC6983525 24325864

[33] Dodhia H , Kun L , Ellis HL , Crompton J , Wierzbicki AS , Williams H , Hodgkinson A , Balazs J . Evaluating quality and its determinants in lipid control for secondary prevention of heart disease and stroke in primary care: a study in an inner London Borough. BMJ Open. 2015;e008678. 10.1136/bmjopen-2015-008678 PMC4679935 26656014

[34] Dreyer R , Arstall M , Tavella R , Morgan C , Weekes A , Beltrame J . Gender differences in patients with stable angina attending primary care practices. Heart Lung Circ. 2011;452–459. 21459670 10.1016/j.hlc.2011.02.005

[35] Forster AS , Dodhia H , Booth H , Dregan A , Fuller F , Miller J , Burgess C , McDermott L , Gulliford MC . Estimating the yield of NHS Health Checks in England: a population‐based cohort study. J Public Health (Bangkok). 2017;234–240. 10.1093/pubmed/fdu079 PMC5942532 25326192

[36] Gulliford MC , Charlton J , Rudd A , Wolfe CD , Toschke AM . Declining 1‐year case‐fatality of stroke and increasing coverage of vascular risk management: population‐based cohort study. J Neurol Neurosurg Psychiatry. 2010;416–422. 20176596 10.1136/jnnp.2009.193136 PMC2921278

[37] Hyun KK , Redfern J , Patel A , Peiris D , Brieger D , Sullivan D , Harris M , Usherwood T , MacMahon S , Lyford M , Woodward M . Gender inequalities in cardiovascular risk factor assessment and management in primary healthcare. Heart. 2017;500–506. 10.1136/heartjnl-2016-310216 28249996

[38] Journath G , Helle M , Petersson U , Theobald H , Nilsson PM ; Hyper‐Q Study Group Sweden . Sex differences in risk factor control of treated hypertensives: a national primary healthcare‐based study in Sweden. Eur J Cardiovasc Prev Rehabil. 2008;258–262. 18525379 10.1097/HJR.0b013e3282f37a45

[39] Majeed A , Moser K , Maxwell R . Age, sex and practice variations in the use of statins in general practice in England and Wales. J Public Health Med. 2000;275–279. 11077897 10.1093/pubmed/22.3.275

[40] Nilsson PM , Theobald H , Journath G , Fritz T . Gender differences in risk factor control and treatment profile in diabetes: a study in 229 Swedish primary health care centres. Scand J Prim Health Care. 2004;27–31. 15119517 10.1080/02813430310003264

[41] Sheppard JP , Fletcher K , Mcmanus RJ , Mant J . Missed opportunities in prevention of cardiovascular disease in primary care. Br J Gen Pract. 2014;e38–e46. 24567581 10.3399/bjgp14X676447 PMC3876174

[42] Turnbull F , Arima H , Heeley E , Cass A , Chalmers J , Morgan C , Patel A , Peiris D , Weekes A , Anderson C . Gender disparities in the assessment and management of cardiovascular risk in primary care: the AusHEART study. Eur J Cardiovasc Prev Rehabil. 2011;498–503. 21450654 10.1177/1741826710389369

[43] Virani SS , Woodard LD , Ramsey DJ , Urech TH , Akeroyd JM , Shah T , Deswal A , Bozkurt B , Ballantyne CM , Petersen LA . Gender disparities in evidence‐based statin therapy in patients with cardiovascular disease. Am J Cardiol. 2015;21–26. 25456865 10.1016/j.amjcard.2014.09.041

[44] Law TK , Yan AT , Gupta A , Kajil M , Tsigoulis M , Singh N , Verma S , Gupta M . Primary prevention of cardiovascular disease: global cardiovascular risk assessment and management in clinical practice. Eur Heart J. 2015;31–36. 29474565 10.1093/ehjqcco/qcv002

[45] Wandell P , Carlsson AC , Holzmann MJ , Arnlov J , Sundquist J , Sundquist K . Association between relevant cardiovascular pharmacotherapies and incident heart failure in patients with atrial fibrillation: a cohort study in primary care. J Hypertens. 2018;1929–1935. 29870433 10.1097/HJH.0000000000001813 PMC6701474

[46] Nanna MG , Wang TY , Xiong Q , Goldberg AC , Robinson JG , Roger VL , Virani SS , Wilson PWF , Louis MJ , Koren A , et al. Sex differences in the use of statins in community practice: patient and provider assessment of lipid management registry. Circ Cardiovasc Qual Outcomes. 2019;e005562. 31416347 10.1161/CIRCOUTCOMES.118.005562 PMC6903404

[47] Owen AJ , Retegan C , Rockell M , Jennings G , Reid CM . Inertia or inaction? Blood pressure management and cardiovascular risk in diabetes. Clin Exp Pharmacol Physiol. 2009;643–647. 19076166 10.1111/j.1440-1681.2008.05125.x

[48] Paulsen MS , Sondergaard J , Reuther L , Larsen PS , Munck AP , Larsen PV , Damsgaard J , Poulsen L , Hansen DG , Jacobsen IA , et al. Treatment of 5413 hypertensive patients: a cross‐sectional study. Fam Pract. 2011;599–607. 21596691 10.1093/fampra/cmr027

[49] Greving JP , Denig P , van der Veen WJ , Beltman FW , Sturkenboom MC , de Zeeuw D , Haaijer‐Ruskamp FM . Does comorbidity explain trends in prescribing of newer antihypertensive agents? J Hypertens. 2004;2209–2215. 15480107 10.1097/00004872-200411000-00025

[50] Hawkins NM , Scholes S , Bajekal M , Love H , O'Flaherty M , Raine R , Capewell S . Community care in England reducing socioeconomic inequalities in heart failure. Circulation. 2012;1050–1057. 22837162 10.1161/CIRCULATIONAHA.111.088047

[51] Murphy NF , Simpson CR , Mcalister FA , Stewart S , Maclntyre K , Kirkpatrick M , Chalmers J , Redpath A , Capewell S , McMurray JJ . National survey of the prevalence, incidence, primary care burden, and treatment of heart failure in Scotland. Heart. 2004;1129–1136. 15367505 10.1136/hrt.2003.029553 PMC1768509

[52] Nilsson PM , Journath G , Palm K , Viigimaa M . Risk factor control in treated hypertensives from Estonia and Sweden. Why the difference? Blood Press. 2009;301–304. 10.1080/08037050701517224 17934917

[53] Law MR , Morris JK , Wald NJ . Use of blood pressure lowering drugs in the prevention of cardiovascular disease: meta‐analysis of 147 randomised trials in the context of expectations from prospective epidemiological studies. BMJ. 2009;b1665. 19454737 10.1136/bmj.b1665 PMC2684577

[54] Smolina K , Ball L , Humphries KH , Khan N , Morgan SG . Sex disparities in post‐acute myocardial infarction pharmacologic treatment initiation and adherence problem for young women. Circ Cardiovasc Qual Outcomes. 2015;586–592. 26462876 10.1161/CIRCOUTCOMES.115.001987

[55] Blomkalns AL , Chen AY , Hochman JS , Peterson ED , Trynosky K , Diercks DB , Brogan GX Jr , Boden WE , Roe MT , Ohman EM , et al.; CRUSADE Investigators . Gender disparities in the diagnosis and treatment of non‐ST‐segment elevation acute coronary syndromes. J Am Coll Cardiol. 2005;832–837. 15766815 10.1016/j.jacc.2004.11.055

[56] Lloyd‐Jones D , Evans JC , Levy D . Hypertension in adults: across the age spectrum. JAMA. 2005;466–472. 16046653 10.1001/jama.294.4.466

[57] Peters SAE , Colantonio LD , Zhao H , Bittner V , Dai Y , Farkouh ME , Monda KL , Safford MM , Muntner P , Woodward M . Sex differences in high‐intensity statin use following myocardial infarction in the United States. J Am Coll Cardiol. 2018;1729–1737. 29673463 10.1016/j.jacc.2018.02.032

[58] Gu Q , Burt VL , Paulose‐ram R , Dillon CF . Gender differences in hypertension treatment, drug utilization patterns, and blood pressure control among us adults with hypertension: data from the National Health and Nutrition Examination Survey 1999–2004. Am J Hypertens. 2008;789–798. 18451806 10.1038/ajh.2008.185

[59] Qvarnstrom M , Wettermark B , Ljungman C , Zarrinhoub R , Hasselstrom J , Manhem K , Sundstrom A , Kahan T . Antihypertensive treatment and control in a large primary care population of 21 167 patients results from the Swedish Primary Care Cardiovascular Database (SPCCD). J Hum Hypertens. 2011;484–491. 20720572 10.1038/jhh.2010.86

[60] Rydberg DM , Mejyr S , Loikas D , Schenck‐Gustafsson K , von Euler M , Malmstrom RE . Sex differences in spontaneous reports on adverse drug events for common antihypertensive drugs. Eur J Clin Pharmacol. 2018;1165–1173. 29804162 10.1007/s00228-018-2480-y PMC6096710

[61] Anand SS , Islam S , Rosengren A , Franzosi MG , Steyn K , Yusufali AH , Keltai M , Diaz R , Rangarajan S , Yusuf S ; INTERHEART investigators . Risk factors for myocardial infarction in women and men: insights from the INTERHEART study. Eur Heart J. 2008;932–940. 18334475 10.1093/eurheartj/ehn018

[62] Kristofferzon M , Lo R , Carlsson M . Myocardial infarction: gender differences in coping and social support. J Adv Nurs. 2003;360–374. 10.1046/j.0309-2402.2003.02815.x 14651708

[63] Mosca L , Mochari‐Greenberger H , Dolor RJ , Newby LK , Robb KJ . Twelve‐year follow‐up of American women's awareness of cardiovascular disease risk and barriers to heart health. Circ Cardiovasc Qual Outcomes. 2010;120–127. 20147489 10.1161/CIRCOUTCOMES.109.915538 PMC2956447

[64] Stringhini S , Spencer B , Marques‐vidal P , Waeber G , Vollenweider P , Paccaud F , Bovet P . Age and gender differences in the social patterning of cardiovascular risk factors in Switzerland: the CoLaus study. PLoS One. 2012;e49443. 23152909 10.1371/journal.pone.0049443 PMC3496703

[65] Jochmann N , Stangl K , Garbe E , Baumann G , Stangl V . Female‐specific aspects in the pharmacotherapy of chronic cardiovascular diseases. Eur Heart J. 2005;1585–1595. 15996977 10.1093/eurheartj/ehi397

[66] Tamargo J , Rosano G , Walther T , Duarte J , Niessner A , Kaski JC , Ceconi C , Drexel H , Kjeldsen K , Savarese G , et al. Gender differences in the effects of cardiovascular drugs. Eur Heart J. 2017;163–182. 10.1093/ehjcvp/pvw042 28329228

[67] Bradley CK , Wang TY , Li S , Robinson JG , Roger VL , Goldberg AC , Virani SS , Louie MJ , Lee LV , Peterson ED , et al. Patient‐reported reasons for declining or discontinuing statin therapy: insights from the PALM registry. J Am Heart Assoc. 2019;e011765 DOI: 10.1161/JAHA.118.011765. 30913959 PMC6509731

[68] Lewey J , Shrank WH , Bowry ADK , Kilabuk E , Brennan TA , Choudhry NK . Gender and racial disparities in adherence to statin therapy: a meta‐analysis. Am Heart J. 2010;665–678.e1. 23622903 10.1016/j.ahj.2013.02.011

[69] Qvamstrom M , Kahan T , Kieler H , Brandt L , Hasselstrom J , Bostrom KB , Manhem K , Hjerpe P , Wettermark B . Persistence to antihypertensive drug classes: a cohort study using Swedish Primary Care Cardiovascular Database (SPCCD). Medicine (Baltimore). 2016;​95:40. 10.1097/MD.0000000000004908 PMC5059050 27749548

