# Supplementary material for: Sex Differences in Cardiovascular Medication Prescription in Primary Care: A Systematic Review and Meta‐Analysis
Source: J Am Heart Assoc. 2020 Jun 20;9(11):e014742. doi: 10.1161/JAHA.119.014742 (PMC7429003; doi:10.1161/JAHA.119.014742)
Supplement: Supplementary file 1 — Tables S1–S11 Figures S1–S11 References 10–52 [file JAH3-9-e014742-s001.pdf]

Supplemental Material

Table S1. Search terms.

	Pubmed	EMBASE	Search names
Primary care	Primary Health Care [Mesh]<br>Primary service [tiab]<br>GP [tiab]<br>Primary Health Care [tiab]<br>Primary healthcare [tiab]<br>Primary medical care [tiab]<br>General practitioner [tiab]<br>General practice [tiab]<br>Family doctor [tiab]<br>Family practitioner [tiab]<br>Family physician [tiab]	(primary adj3 care*).tw.<br>primary service*.tw.<br>GP.tw.<br>General practice*.tw.<br>Primary health?care.tw.<br>exp primary medical care/<br>exp general practitioner/<br>exp general practice/<br>(family adj (doctor or practitioner or physician)).tw.	Primary care v1<br>Primary care v2
CVD risk scores	Cardiovascular score [tiab]<br>Cardiovascular risk score [tiab]<br>ASSIGN score [tiab]<br>Qrisk [tiab]<br>Systematic Coronary Risk Evaluation [tiab]<br>Framingham score [tiab]<br>Framingham risk [tiab]<br>Framingham index [tiab]<br>Pooled cohort equation [tiab]	Exp cardiovascular risk/<br>(cardiovascular adj2 score).tw.<br>(assign adj score).tw.<br>QRisk.tw.<br>Systematic Coronary Risk Evaluation.tw.<br>(Framingham adj4 (score or risk or index)).tw.<br>pooled cohort equation.tw.	Cvd risk scores v1<br>*risk factor will go in risk factor section.
Primary prevention	Primary prevention [MeSH]<br>Primary prevention [tiab]	exp primary prevention/<br>(primary adj2 prevention).tw.	Primary prevention v1<br>Primary prevention v2
Secondary prevention	Secondary prevention [MeSH]<br>Secondary prevention [tiab]	exp secondary prevention/<br>(secondary adj2 prevention).tw.	Secondary prevention v1<br>Secondary prevention v2

	Pubmed	EMBASE	Search names
Sex	Male[MeSH]<br>Male[tiab]<br>Men[tiab]<br>Man[tiab]<br>Female[MeSH]<br>Female[tiab]<br>Women[tiab]<br>Woman[tiab]<br>Sex[MeSH]<br>Sex[tiab]<br>Gender[tiab]	male/<br>(mean or man or male).tw.<br>female/<br>(woman or women or female).tw.<br>gender/<br>sex/<br>(gender* or sex*).tw.	Men and women v2<br>Sex gender v2
Risk assess	Risk factors[MeSH]<br>Risk factors [tiab]<br>Risk assessment [MeSH]<br>Risk assessment [tiab]<br>Absolute risk [tiab]<br>Health screen [tiab]<br>Health screening [tiab]<br>Health measurement [tiab]<br>Health assessment [tiab]<br>Health care disparity [MeSH]<br>Health care disparity [tiab]<br>Health care disparities [tiab]	Exp risk factor/<br>Exp risk assessment/<br>(risk adj5 (assess* or measure* or screem*)).tw.<br>(absolute adj5 risk*).tw.<br>exp health care disparity/<br>(health? Care adj3 disparit*).tw.	Risk assess v2<br>Risk assess v4
Drugs	(statin* or lipid lowering).tw.<br>exp hydroxymethylglutaryl coenzyme A reductase inhibitor/<br>((blood pressure adj3 medication*) or	cardiovascular drugs/therapeutic use [Mesh]<br>cardiovascular diseases/therapy [mesh]<br>Hydroxymethylglutaryl-CoA Reductase Inhibitors [Mesh]	standalone:<br>combined with drugs tab:<br>all drug terns and meds v2<br>same as angiotensin II receptor

	Pubmed	EMBASE	Search names
	blood pressure lowering or bp?lowering).tw.<br>exp antihypertensive agent/<br>(angiotensin II receptor blocker* or ARB*).tw.<br>(angiotensin?converting enzyme inhibitor* or ACE* or ACEI* or ACEi*).tw.<br>exp dipeptidyl carboxypeptidase inhibitor/<br>(beta blocker* or b?blocker*).tw.<br>exp beta adrenergic receptor blocking agent/<br>antiplatelet.tw.<br>exp antithrombocytic agent/<br>aspirin.tw<br>antithrombotic*.tw<br>exp nonsteroid antiinflammatory agent/<br>((calcium?channel and (blocker* or blocking)) or (calcium adj2 antagonist*) or calcium?antagonist* or CCB*).tw.<br>exp calcium channel blocking agent/<br>exp diuretic agent/<br>diuretic*.tw.	statin [tiab]<br>statins [tiab]<br>lipid lowering [tiab]<br>blood pressure medication [tiab]<br>blood pressure lowering [tiab]<br>bp lowering [tiab]<br>antihypertensive agent [Mesh]<br>antihypertensive [tiab]<br>Angiotensin Receptor Antagonists [Mesh]<br>Angiotensin Receptor Antagonist [tiab]<br>Angiotensin Receptor Antagonists [tiab]<br>angiotensin II receptor blocker [tiab]<br>angiotensin II receptor blockers [tiab]<br>angiotensin 2 receptor blocker [tiab]<br>angiotensin 2 receptor blockers [tiab]<br>ARB[tiab]<br>ARBs[tiab]<br>Angiotensin Converting Enzyme Inhibitors [Mesh]<br>Angiotensin Converting Enzyme Inhibitor [tiab]<br>Angiotensin Converting Enzyme Inhibitors [tiab]<br>ACE inhibitor [tiab]<br>ACE inhibitors [tiab]<br>ACEi [tiab]<br>Adrenergic beta-Antagonists [Mesh]	blocker [mesh]<br>CVD meds v2

	Pubmed	EMBASE	Search names
		beta blocker [tiab]<br>beta blockers [tiab]<br>b blocker [tiab]<br>b blockers [tiab]<br>Anti-Inflammatory Agents, Non-Steroidal [Mesh]<br>antithrombotic [tiab]<br>antithrombotics [tiab]<br>antiplatelet [tiab]<br>aspirin [MeSH]<br>aspirin [tiab]<br>Calcium Channel Blockers [MeSH]<br>Calcium Channel Blocker [tiab]<br>Calcium Channel Blockers [tiab]<br>calcium antagonist [tiab]<br>calcium antagonists [tiab]<br>CCB [tiab]<br>CCBs [tiab]<br>diuretics [Mesh]<br>diuretic [tiab]<br>diuretics [tiab]	

Table S2. Data extraction form.

Study (author)		
Publication year		
Source	Study ID (Corresponding with reference software)	
	Reviewer ID (MZ, EM, or KH)	
Study design	Study type	
Study characteristics	Year of study	
	Performed country	
Patient characteristics	CVD status	
	Prevention type	
	Age	
	Women	
	Mean women	
	Men	
	Mean men	
Aspirin	Study sample	
	Study women	
	Number of women on medications	
	Percentage of women on medications	
	Number of men on medications	
	Percentage of women on medications	
	Differences (women-men)	
	Women-to-men prevalence ratio	
	Maximum adjustment available	
Statins	Study sample	
	Study women	
	Number of women on medications	
	Percentage of women on medications	
	Number of men on medications	
	Percentage of women on medications	
	Differences (women-men)	
	Women-to-men prevalence ratio	
	Maximum adjustment available	
Beta blockers	Study sample	
	Study women	
	Number of women on medications	
	Percentage of women on medications	
	Number of men on medications	
	Percentage of women on medications	

Study (author)		
	Differences (women-men)	
	Women-to-men prevalence ratio	
	Maximum adjustment available	
Calcium channel blockers	Study sample	
	Study women	
	Number of women on medications	
	Percentage of women on medications	
	Number of men on medications	
	Percentage of women on medications	
	Differences (women-men)	
	Women-to-men prevalence ratio	
	Maximum adjustment available	
ACE-inhibitors	Study sample	
	Study women	
	Number of women on medications	
	Percentage of women on medications	
	Number of men on medications	
	Percentage of women on medications	
	Differences (women-men)	
	Women-to-men prevalence ratio	
	Maximum adjustment available	
Diuretics	Study sample	
	Study women	
	Number of women on medications	
	Percentage of women on medications	
	Number of men on medications	
	Percentage of women on medications	
	Differences (women-men)	
	Women-to-men prevalence ratio	
	Maximum adjustment available	
Key findings		

Table S3. Quality assessment tool: Newcastle-Ottawa Scale.

Selection: (Maximum 3 stars)

1) Representativeness of the sample:

a) Truly representative of the average in the target population. * (all subjects or random sampling)

b) Somewhat representative of the average in the target population. * (non-random sampling)

c) Selected group of users.

d) No description of the sampling strategy.

2) Sample size:

a) Justified and satisfactory. *

b) Not justified.

3) Non-respondents:

a) Comparability between respondents and non-respondents characteristics is established, and the response rate is satisfactory. *

b) The response rate is unsatisfactory, or the comparability between respondents and non-respondents is unsatisfactory.

c) No description of the response rate or the characteristics of the responders and the non-responders.

Comparability: (Maximum 2 stars)

1) The subjects in different outcome groups are comparable, based on the study design or analysis. Confounding factors are controlled.

a) The study controls for the most important factor (age). *

b) The study control for any additional factor. *

Outcome: (Maximum 3 stars)

1) Assessment of the outcome:

a) Independent blind assessment. **

b) Record linkage. **

c) Self report. *

d) No description.

2) Statistical test:

a) The statistical test used to analyze the data is clearly described and appropriate, and the measurement of the association is presented, including confidence intervals and the probability level (p value). *

b) The statistical test is not appropriate, not described or incomplete.

Studies with more than four stars will be counted as satisfactory and thus can be included in systematic review.

Table S4. Quality assessment.

Study		Selection (3)			Comparability (2)	Outcome(3)		Total
Study	Year	Representativeness	Sample size	Non-respondent	Adjustment	Outcome	Statistical test	
Carlsson A.C. et al[21]	2012	1	1	1	1	2	1	7
Carroll K et al[22]	2003	1	1	1	1	2	1	7
Catalan-Ramos A et al[32]	2014	1	1	1	0	2	1	6
Al-Lawati J.A. et al[10]	2012	1	1	1	0	2	1	6
Crilly M et al[23]	2007	1	1	1	2	2	1	8
Dodhia H et al[33]	2015	1	1	1	2	2	1	8
Dreyer R et al[34]	2009	1	1	1	2	2	1	8
Driscoll A. et al[24]	2011	1	1	1	2	2	1	8
Emberson J.R. et al[25]	2005	1	1	1	2	2	1	8
Forster A.S. et al[35]	2014	1	1	1	0	2	1	6
Greving J.P. et al[49]	2004	1	1	1	2	2	1	8
Gulliford M.C. et al[36]	2010	1	1	1	2	2	1	8
Hawkins N.M. et al[50]	2012	1	1	1	2	2	1	8
Hendrix K.H. et al[26]	2005	1	1	1	0	2	0	5
Hippisley-Cox J et al[27]	2001	1	1	1	2	2	1	8
Hyun K. et al[37]	2012	1	1	1	2	2	1	8
Journath G. et al[38]	2008	1	1	1	1	2	1	7
Brady A.J.B. et al[20]	2005	1	1	1	0	2	0	5
Weler D.J. et al[18]	2005	1	1	1	2	2	1	8
Paulsen M.S. et al[48]	2011	1	1	1	2	2	1	8
Lahoz C. et al[12]	2009	1	1	1	2	2	1	8

Study		Selection (3)			Comparability (2)	Outcome(3)		Total
Study	Year	Representativeness	Sample size	Non-respondent	Adjustment	Outcome	Statistical test	
Sheppard J.P. et al[41]	2014	1	1	1	0	2	0	5
Svilaas A et al[16]	2000	1	1	1	0	2	1	6
Tabenkin H et al[17]	2010	1	1	1	2	2	1	8
Turnbull F et al[42]	2010	1	1	1	1	1	1	6
Virani S.S. et al[43]	2011	1	1	1	2	2	1	8
Majeed A. et al[39]	2000	1	1	1	0	2	0	5
Majeed A. et al[14]	2005	1	1	1	0	2	0	5
Murphy N. et al[51]	2004	1	1	1	2	2	1	8
Nilsson P.M. et al[52]	2007	1	1	1	0	2	1	6
Nilsson P.M. et al[40]	2004	1	1	1	1	2	1	7
Owen A. et al[47]	2009	1	1	1	2	0	1	6
Lawlor D.A. et al[29]	2004	1	1	1	2	2	1	8
Bull N et al[31]	2003	1	1	1	2	2	1	8
Macchia A et al[13]	2012	1	1	1	2	2	1	8
Qato D.M et al[15]	2016	1	1	1	2	1	1	7
Saposnik G. et al[30]	2009	1	1	1	0	1	1	5
Brady A.J. et al[20]	2001	1	1	1	0	2	0	5
Alberts M.J. et al[28]	2009	1	1	1	0	2	1	6
Lee C. et al[19]	2019	1	1	1	2	2	1	8
Wandell P. et al[45]	2018	1	1	1	1	2	1	7
Law T.K. et al[44]	2015	1	1	1	0	1	1	5

Table S5. Sex difference on aspirin prescription.

Study, year	CVD status	Age	Age of women	Age of men	% for women	% for men	Unadjusted PR	Adjusted PR¶
Brady, 1998[11]	CVD	67	NA	NA	46%	53%	0.86 (0.83-0.88)	0.88 (0.81-0.95)
Macchia, 2005[13]	CVD	NA	NA	NA	78%	85%	0.92 (0.91-0.93)	NA
Qato D.M, 2011[15]	Mixed (CVD+High-risk**)	52	NA	NA	60%	63%	0.96 (0.91-1.01)	0.84 (0.71-0.98)
Al-Lawati, 2012*[10]	Mixed (CVD+DM)	54	54	54	67%	67%	1.00 (0.95-1.06)	0.85 (0.74-0.97)
Brady, 2002[20]	CVD	67	NA	NA	74%	80%	0.92 (0.90-0.94)	0.88 (0.81-0.95)
Carlsson, 2002[21]	CVD	76	75	74	35%	32%	1.09 (1.02-1.16)	0.91 (0.79-1.04)
Carroll, 2001[22]	CVD	NA	NA	NA	59%	65%	0.90 (0.87-0.94)	NA
Crilly, 2001[23]	CVD	69	NA	NA	81%	86%	0.94 (0.89-0.99)	0.89 (0.81-0.97)
Driscoll, 2007[24]	CVD	73	74	72	71%	80%	0.89 (0.87-0.91)	0.90 (0.80-1.01)
Emberson, 2001[25]	Mixed (CVD+DM)	NA	NA	NA	14%	27%	0.51 (0.47-0.56)	NA
Hendrix, 2005*[26]	Mixed (CVD+HTN)	NA	NA	NA	15%	39%	0.38 (0.37-0.39)	NA
Hippisley-Cox, 2001*[27]	Mixed, (CVD+High-risk**)	NA	NA	NA	71%	76%	0.94 (0.91-0.97)	NA
Weler, 2003[18]	Mixed (CVD+DM)	65	66	63	46%	57%	0.80 (0.75-0.85)	0.88 (0.81-0.94)
Lahoz, 2008*[12]	CVD	65	NA	NA	55%	64%	0.87 (0.83-0.90)	0.88 (0.82-0.94)
Majeed, 2002[14]	CVD	NA	NA	NA	59%	66%	0.89 (0.83-0.95)	NA
Svilaas, 1997[16]	CVD	69	NA	NA	47%	58%	0.81 (0.74-0.89)	0.89 (0.82-0.97)
Tabenkin, 2004[17]	Mixed (CVD+HTN)	53	52	53	35%	55%	0.64 (0.51-0.80)	0.84 (0.73-0.98)
Lee, 2018[19]	CVD	67	65	68	41%	57%	0.72 (0.71, 0.73)	0.88 (0.81, 0.95)
Pooled		64	67	65	41%	56%	0.81 (0.73-0.92)	0.87 (0.81-0.94)

CVD: cardiovascular disease; PR: prevalence ratio; NA: not available; %: percentage of using medication; mixed: patients at high-risks and with established cardiovascular disease; DM: diabetes; HTN: hypertension

*Publication year

** No high-risk assessment tool is available

¶ Mean age of study population in each study was adjusted.

Table S6. Sex difference on statin prescription.

Study, year	CVD status	Age	Age of Women	Age of men	% for women	% for men	Unadjusted PR	Adjusted PR ¶
Alberts, 2004[28]	CVD	69	NA	NA	66%	69%	0.96 (0.94-0.97)	0.87 (0.82-0.92)
Brady, 1998[11]	CVD	69	NA	NA	13%	18%	0.73 (0.69-0.78)	0.87 (0.82-0.92)
Lawlor, 2000[29]	CVD	60-79	NA	NA	27%	24%	1.12 (0.93-1.36)	NA
Macchia, 2005[13]	CVD	68.1	74	65	67%	80%	0.84 (0.83-0.86)	0.88 (0.83-0.93)
Qato, 2011[15]	High-risk**	52.2	NA	NA	25%	25%	1.00 (0.90-1.11)	1.05 (0.94-1.18)
Saposnik, 2004[30]	CVD	67	NA	NA	79%	78%	1.01 (0.95-1.08)	0.89 (0.84-0.93)
Brady, 2002[20]	CVD	67	NA	NA	45%	52%	0.87 (0.83-0.90)	0.89 (0.84-0.93)
Bull, 2003[31]	CVD	>40	NA	NA	21%	28%	0.75 (0.70-0.79)	NA
Carlsson, 2002[21]	CVD	75.5	75	74	18%	24%	0.78 (0.71-0.85)	0.81 (0.73-0.89)
Carroll K, 2001[22]	CVD	>44	NA	NA	38%	49%	0.77 (0.73-0.82)	NA
Catalan-Ramos, 2009[32]	High-risk, defined by FRS	51	NA	NA	71%	70%	1.01 (1.00-1.02)	1.07 (0.94, 1.20)
Crilly, 2001[23]	CVD	69	71	67	53%	56%	0.93 (0.84-1.04)	0.87 (0.82-0.92)
Dodhia, 2013[33]	CVD	70	NA	NA	75%	83%	0.90 (1.03-1.09)	0.86 (0.81-0.91)
Dreyer, 2007[34]	CVD	70	NA	NA	76%	85%	0.89 (0.85-0.94)	0.86 (0.81-0.91)
Emberson, 2001[25]	Mixed (CVD+DM)	60-79	NA	NA	8%	7%	1.10 (0.95-1.29)	NA
Forster, 2013[35]	High-risk, NHS health check	40-74	NA	NA	21%	18%	1.21 (1.14-1.29)	NA
Gulliford, 2010[36]	CVD	73	NA	NA	16%	19%	0.85 (0.79-0.92)	0.83 (0.77-0.90)
Hendrix, 2005*[26]	Mixed (CVD+HTN)	62	NA	NA	29%	41%	0.70 (0.69-0.72)	0.94 (0.89-1.00)
Hyun, 2012[37]	Mixed (CVD+high risk defined by FRS)	61	NA	NA	66%	68%	0.97 (0.95-0.99)	0.95 (0.90-1.01)

Study, year	CVD status	Age	Age of Women	Age of men	% for women	% for men	Unadjusted PR	Adjusted PR ¶
Journath, 2005[38]	High-risk (HTN)	66	67	65	28%	33%	0.85 (0.79-0.92)	0.90 (0.85-0.94)
Lahoz, 2008*[12]	CVD	65	68	65	77%	80%	0.96 (0.93-0.98)	0.91 (0.86-0.95)
Majeed, 1996[39]	CVD	NA	NA	NA	8%	13%	0.62 (0.59-0.65)	NA
Nilsson, 2009[52]	Mixed (CVD+HTN))	65	NA	NA	96%	92%	1.04 (1.03-1.05)	0.91 (0.86-0.95)
Sheppard, 2009[41]	Mixed (CVD+High-risk defined by FRS)	54	NA	NA	92%	72%	1.27 (1.24-1.31)	1.03 (0.93-1.14)
Turnbull, 2008[42]	Mixed (CVD+ high-risk defined by FRS)	68	68	68	53%	59%	0.90 (0.85-0.95)	0.88 (0.83-0.93)
Virani, 2011[43]	CVD	71	66	71	58%	65%	0.89 (0.88-0.90)	0.85 (0.79-0.91)
Law, 2010[44]	High-risk, defined by FRS	58	NA	NA	91%	93%	0.97 (0.91-1.04)	0.98 (0.91-1.06)
Lee, 2018[19]	CVD	67	65	68	56%	75%	0.74 (0.73, 0.74)	0.89 (0.84, 0.93)
Wandell, 2007[45]	CVD	NA	NA	NA	33%	39%	0.84 (0.79, 0.89)	NA
Nanna, 2015[46]	Mixed (CVD+High-risk**)	68	68	68	67%	78%	0.85 (0.83, 0.88)	0.88 (0.83-0.93)
Pooled		65	71	68	60%	63%	0.90 (0.85, 0.95)	0.91 (0.87-0.95)

CVD: cardiovascular disease; PR: prevalence ratio; NA: not available; %: percentage of using medication; mixed: patients at high-risks and with established CVD; FRS: Framingham risk score; HTN: hypertension; DM: diabetes

*Publication year

**No cardiovascular risk assessment tool is available

¶ Mean age of study population in each study was adjusted.

Table S7. Sex difference on beta-blockers prescription.

Study, year	CVD status	Age	Age of women	Age of men	% for women	% for men	Unadjusted PR	Adjusted PR ¶
Brady, 1998[11]	CVD	69	NA	NA	19%	23%	0.86 (0.81-0.90)	0.91 (0.85-0.97)
Macchia, 2003[13]	CVD	68	74	65	64%	68%	0.93 (0.91-0.95)	0.92 (0.86-0.97)
Al-Lawati, 2007[10]	Mixed (CVD+DM)	54	54	54	7%	7%	1.08 (0.73-1.59)	1.00 (0.88-1.14)
Brady, 2002[20]	CVD	67	NA	NA	38%	42%	0.91 (0.87-0.95)	0.92 (0.87-0.98)
Carlsson, 2013[21]	CVD	76	75	74	59%	55%	1.07 (1.03-1.11)	0.87 (0.77-0.98)
Carroll, 2001[22]	CVD	>44	NA	NA	20%	22%	0.91 (1.09-1.10)	NA
Catalan-Ramos, 2009[32]	High-risks, defined by FRS	51	NA	NA	40%	36%	1.09 (1.08-1.10)	1.02 (0.87-1.19)
Crilly M, 2001[23]	CVD	69	71	67	28%	38%	0.74 (0.63-0.88)	0.91 (0.85-0.97)
Dreyer, 2001[34]	CVD	70	NA	NA	51%	55%	0.93 (0.85-1.01)	0.90 (0.84-0.97)
Emberson, 2001[25]	Mixed (CVD+DM)	60-79	NA	NA	25%	17%	1.49 (1.37-1.62)	NA
Greving, 2000[49]	High-risks (HTN)	63	NA	NA	41%	41%	1.01 (0.95-1.07)	0.94 (0.89-1.00)
Hawkins,2007[50]	CVD	68	NA	NA	24%	28%	0.86 (0.82-0.89)	0.92 (0.86-0.97)
Hendrix, 2005*[26]	Mixed (CVD+HTN)	62	NA	NA	28%	32%	0.86 (0.84-0.88)	0.95 (0.89-1.01)
Hippisley-Cox, 2001*[27]	CVD	62	NA	NA	49%	51%	0.96 (0.91=1.01)	0.95 (0.89-1.01)
Journath, 2005[38]	High-risks (HTN)	66	67	65	54%	51%	1.05 (1.01-1.11)	0.93 (0.88-0.98)
Lahoz, 2008*[12]	CVD	65	68	65	41%	49%	0.83 (0.78-0.87)	0.93 (0.88-0.99)
Murphy,2004[51]	High-risks (HTN)	NA	NA	NA	20%	23%	0.86 (0.68-1.09)	NA
Owen, 2009[47]	High-risks (DM)	63	63	62	19%	19%	0.99 (0.92-1.06)	0.94 (0.89-1.00)
Paulsen, 2011[48]	High-risks (HTN)	66	66	66	29%	28%	1.04 (0.96-1.13)	0.98 (0.90-1.06)
Lee, 2018[19]	CVD	67	65	68	38%	50%	0.76 (0.75,0.77)	0.93 (0.88, 0.98)
Wandell, 2007[45]	CVD	NA	NA	NA	79%	75%	1.05 (1.02, 1.07)	NA
Pooled		65	69	66	38%	38%	0.95 (0.89, 1.02)	0.93 (0.88, 0.99)

CVD: cardiovascular disease; PR: prevalence ratio; NA: not available; %: percentage of using medication; DM: diabetes; FRS: Framingham risk score; HTN: hypertension

*Publication year

¶ Mean age of study population in each study was adjusted.

Table S8. Sex difference on calcium channel blockers prescription.

Study, year	CVD status	Age	Age of women	Age of men	% for women	% for men	Unadjusted PR	Adjusted PR ¶
Brady, 1998[11]	CVD	69	NA	NA	12%	14%	0.85 (0.80-0.91)	0.98 (0.87-1.13)
Al-Lawati, 2007[10]	High-risks (DM)	54	54	54	2%	1%	1.38 (0.58-3.27)	0.87 (0.72-1.04)
Carlsson, 2013[21]	CVD	75	75	74	7%	6%	1.17 (0.97-1.40)	1.04 (0.85-1.29)
Catalan-Ramos, 2009[32]	High-risks (HTN)	51	NA	NA	25%	27%	0.94 (0.93-0.95)	0.85 (0.67-1.05)
Dreyer, 2007[34]	CVD	70	NA	NA	39%	31%	1.26 (1.11-1.42)	1.00 (0.86-1.16)
Greving, 2000[49]	High-risks (HTN)	63	NA	NA	18%	24%	0.74 (0.68-0.81)	0.94 (0.85-1.04)
Hendrix, 2005*[26]	Mixed (CVD+HTN)	62	NA	NA	30%	28%	1.08 (1.05-1.10)	0.93 (0.84-1.03)
Journath, 2005[38]	High-risks (HTN)	66	67	65	26%	34%	0.78 (0.72-0.84)	0.96 (0.86-1.07)
Lahoz, 2008*[12]	CVD	65	68	65	27%	24%	1.13 (1.04-1.23)	1.18 (1.06-1.31)
Nilsson, 2007*[52]	High-risks (HTN)	52	53	51	26%	34%	0.77 (0.64-0.92)	0.68 (0.53-0.89)
Owen, 2009[47]	High-risks (DM+HTN)	63	63	62	26%	27%	0.96 (0.90-1.02)	0.94 (0.87-1.02)
Paulsen, 2011[48]	High-risks (HTN)	66	66	66	32%	38%	0.84 (0.78-0.90)	0.61 (0.55-0.69)
Wandell, 2007[45]	CVD	NA	NA	NA	37%	34%	1.07 (1.01, 1.14)	NA
Pooled		63	65	64	25%	26%	0.95 (0.87-1.05)	0.94 (0.85-1.04)

CVD: cardiovascular disease; PR: prevalence ratio; NA: not available; %: percentage of using medication; HTN: hypertension; DM: diabetes

*Publication year

¶ Mean age of study population in each study was adjusted.

Table S9. Sex difference on ACE-inhibitors prescription.

Author, year	CVD status	Age	Age of women	Age of men	% for women	% for men	Unadjusted PR	Adjusted PR ¶
Brady , 1998[11]	CVD	69	NA	NA	12%	14%	0.85 (0.80-0.91)	0.84 (0.79-0.89)
Macchina, 2011[13]	CVD	68	74	65	79%	79%	1.00 (0.98-1.01)	0.81 (0.66-0.99)
Al-Lawati, 2007[10]	High-risks (DM)	54	54	54	37%	46%	0.80 (0.71-0.92)	0.74 (0.57-0.96)
Brady, 2002[20]	CVD	67	NA	NA	25%	28%	0.89 (0.84-0.95)	0.81 (0.67-0.97)
Catalan-Ramos, 2009[32]	High-risks (HTN)	51	NA	NA	53%	59%	0.90 (0.89, 0.90)	0.73 (0.52-1.01)
Dreyer, 2007[34]	CVD	70	NA	NA	44%	52%	0.85 (0.77-0.93)	0.82 (0.65-1.05)
Emberson, 2001[25]	Mixed (CVD+DM)	60-79	NA	NA	26%	23%	1.11 (1.03-1.20)	NA
Greving, 2000[49]	High-risks (HTN)	63	NA	NA	28%	37%	0.76 (0.71-0.82)	0.78 (0.67-0.91)
Hawkins, 2007[50]	CVD	68	NA	NA	52%	56%	0.92 (0.90-0.94)	0.81 (0.66-0.99)
Hendrix, 2005*[26]	Mixed (CVD+HTN)	62	NA	NA	44%	52%	0.85 (0.84-0.87)	0.78 (0.67-0.91)
Journath, 2005[38]	High-risks (HTN)	66	67	65	18%	27%	0.67 (0.61-0.73)	0.80 (0.67-0.95)
Lahoz, 2008*[12]	CVD	65	68	65	40%	39%	1.01 (0.95-1.07)	0.80 (0.68-0.94)
Majeed, 2002[14]	CVD	NA	NA	NA	68%	76%	0.89 (0.85-0.94)	NA
Murphy, 2004[51]	CVD	NA	NA	NA	34%	46%	0.74 (0.64-0.87)	NA
Nilsson, 2007*[52]	High-risks (HTN)	52	53	51	18%	27%	0.67 (0.53-0.83)	0.73 (0.54-0.99)
Nilsson, 2004[40]	Mixed (CVD+DM)	65	NA	NA	27%	33%	0.80 (0.76-0.86)	0.80 (0.68-0.94)
Owen, 2009[47]	High-risks (DM+HTN)	62	63	62	45%	51%	0.87 (0.84-0.91)	0.78 (0.67-0.91)
Paulsen, 2011[48]	High-risks (HTN)	66	66	66	37%	48%	0.77 (0.72-0.82)	0.80 (0.67-0.95)
Tabenkin, 2004[17]	High-risks (HTN)	53	52	53	41%	52%	0.79 (0.64-0.98)	0.73 (0.55-0.98)
Lee, 2018[19]	CVD	67	65	68	55%	69%	0.80 (0.80, 0.81)	0.80 (0.68, 0.95)
Wandell, 2007[45]	CVD	NA	NA	NA	33%	40%	0.83 (0.78, 0.88)	NA
Pooled		63	65	61	51%	57%	0.85 (0.81, 0.89)	0.84 (0.79, 0.89)

CVD: cardiovascular disease; PR: prevalence ratio; NA: not available; %: percentage of using medication; mixed: patients at high-risks and with established CVD; DM: diabetes; HTN: hypertension

*Publication year

¶ Mean age of study population in each study was adjusted.

Table S10. Sex difference on diuretics prescription.

Author, year	CVD status	Age	Age of women	Age of men	% for women	% for men	Unadjusted PR	Adjusted PR ¶
Brady, 1998[11]	CVD	69	NA	NA	29%	19%	1.51 (1.42-1.61)	1.32 (1.20-1.44)
Al-Lawati, 2012[10]	High-risks (DM)	54	54	54	4%	4%	1.16 (0.68-2.01)	1.33 (1.17-1.53)
Carlsson, 2013[21]	CVD	76	76	75	56%	42%	1.33 (1.27-1.40)	1.30 (1.10-1.55)
Catalan-Ramos, 2009[32]	High-risks (HTN)	51	NA	NA	68%	48%	1.43 (1.42-1.44)	1.34 (1.14-1.58)
Greving, 2009[49]	High-risks (HTN)	63	NA	NA	47%	32%	1.48 (1.39, 1.58)	1.32 (1.22-1.43)
Hendrix, 2005*[26]	Mixed (CVD+HTN)	62	NA	NA	58%	50%	1.14 (1.13-1.16)	1.32 (1.22-1.44)
Journath, 2005[38]	High-risks (HTN)	66	67	65	64%	48%	1.35 (1.29-1.41)	1.32 (1.21-1.44)
Lahoz, 2008*[12]	CVD	65	68	65	46%	32%	1.43 (1.35-1.52)	1.31 (1.21-1.43)
Majeed, 2002[14]	CVD	NA	NA	NA	81%	79%	1.02 (0.98-1.07)	NA
Murphy, 2004[51]	CVD	NA	NA	NA	81%	80%	1.01 (0.95-1.07)	NA
Nilsson, 2007*[52]	High-risks (HTN)	52	53	51	59%	45%	1.31 (1.16-1.48)	1.37 (1.15-1.56)
Owen, 2009[47]	High-risks (DM+HTN)	63	63	62	23%	17%	1.34 (1.25-1.44)	1.32 (1.22-1.43)
Paulsen, 2011[48]	High-risks (HTN)	66	66	66	66%	57%	1.01 (0.97-1.05)	1.32 (1.20-1.44)
Wandell, 2007[45]	CVD	NA	NA	NA	69%	54%	1.28 (1.23, 1.32)	NA
Pooled		63	65	64	47%	39%	1.26 (1.17, 1.37)	1.32 (1.22-1.43)

CVD: cardiovascular disease; PR: prevalence ratio; NA: not available; %: percentage of using medication; Mixed: patients at high-risks and with established CVD; HTN: hypertension; DM: diabetes

*Publication year

¶ Mean age of study population in each study was adjusted.

Table S11. Inclusion information, stratified by CVD status.

		Aspirin	Statin	BB	CCB	ACE-Inhibitor	Diuretics
High-risk	No. Paper	NA	7	7	7	8	7
	No. Women	NA	399,002	343,724	343,866	344,046	343,866
	No. Men	NA	406,962	344,523	344,504	344,726	344,204
	PP. women	NA	67%	39%	25%	52%	70%
	PP. men	NA	64%	36%	27%	58%	47%
	Pooled PR	NA	0.93(0.82,1.07)	1.04(0.96,1.12)	0.85(0.61,1.18)	0.79(0.49,1.26)	1.31(0.77,2.20)
CVD	No. Paper	11	19	12	5	10	6
	No. Women	98,294	170,702	111,640	19,733	104,151	15,364
	No. Men	130,704	1,177,332	140,974	30,903	130,772	24,027
	PP. women	48%	44%	38%	18%	50%	52%
	PP. men	62%	63%	47%	19%	58%	36%
	Pooled PR	0.89(0.84,0.94)	0.85(0.80,0.90)	0.90(0.85,0.96)	1.08(0.94,1.23)	0.88(0.84,0.93)	1.25(1.09,1.43)
Mixed	No. Paper	7	5	2	NA	3	NA
	No. Women	42,025	18,552	33,494	NA	37,787	NA
	No. Men	55,855	21,018	47,552	NA	52,634	NA
	PP. women	24%	61%	28%	NA	40%	NA
	PP. men	42%	61%	31%	NA	47%	NA
	Pooled PR	0.71(0.54,0.93)	1.05(0.93,1.18)	1.13(0.66,1.94)	NA	0.91(0.75,1.10)	NA

PP: Pooled prevalence; PR: prevalence ratio; CCB: calcium channel blocker; NA: not available

Figure S1. Women-to-men prevalence ratio from 10 studies reporting all four antihypertensive medications.

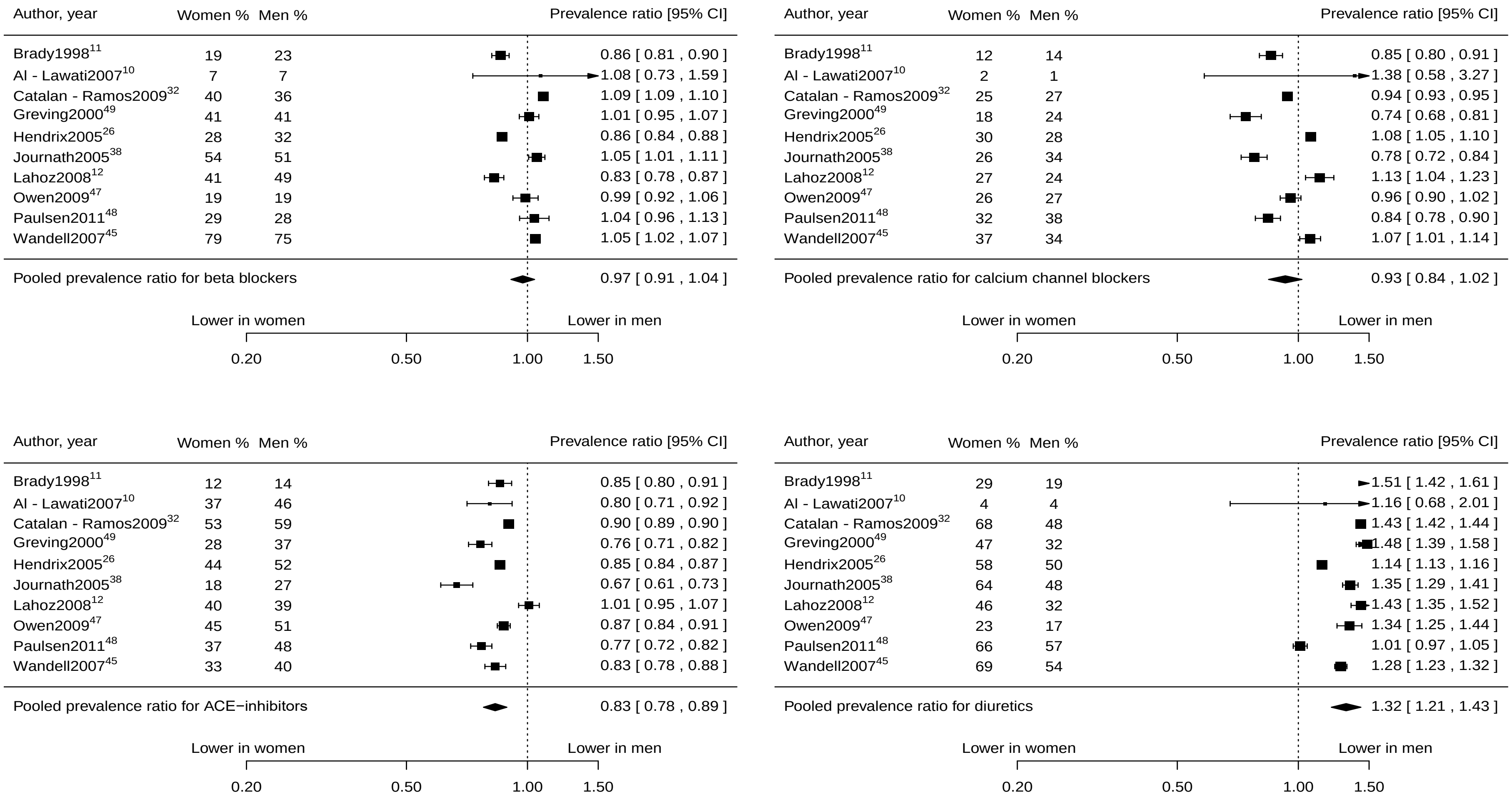

For each study, the square is centered on the women-to-men prevalence ratio and the horizontal lines show the associated 95% confidence interval. The diamond indicates the pooled summary and its 95% confidence interval.

Figure S2. Association between age and sex differences in the prescription of cardiovascular medication.

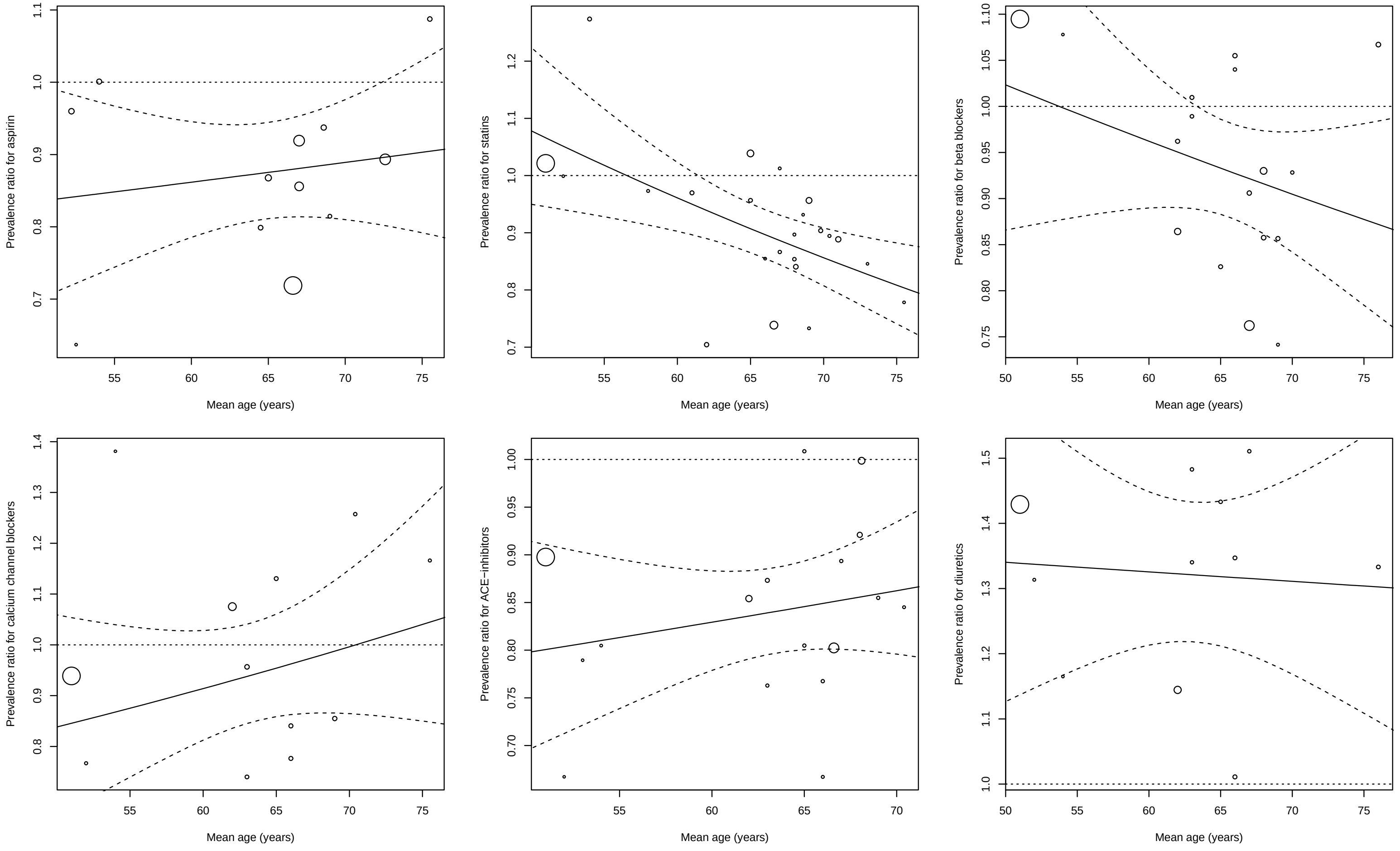

Bubbles are individual studies; diameters of the bubbles are proportional to studies weight for analysis.

Figure S3. Association between age difference between the sexes and sex differences in the prescription of cardiovascular medication.

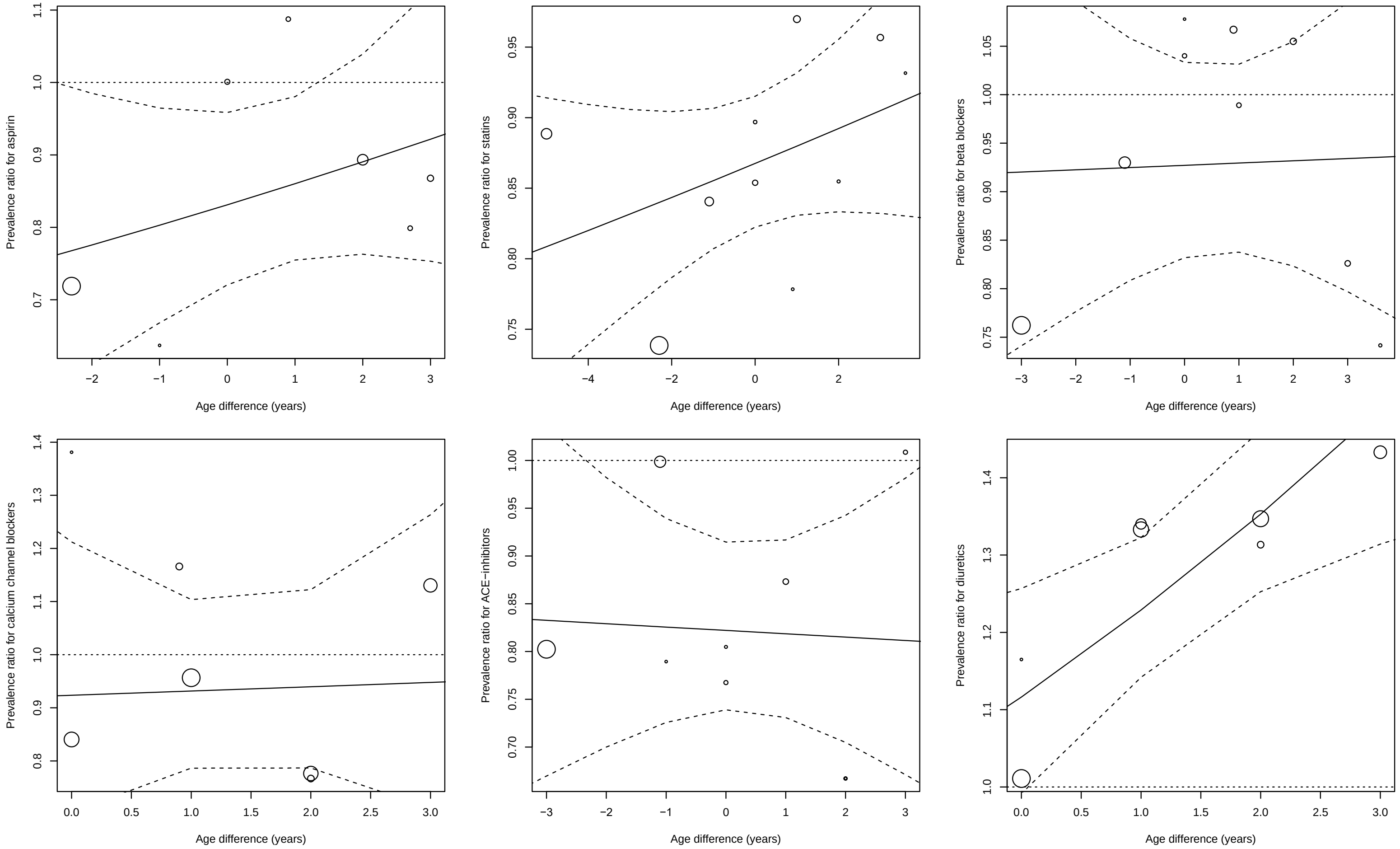

Bubbles are individual studies; diameters of the bubbles are proportional to studies weight for analysis.

Figure S4. Yearly trend of sex differences in the prescription of cardiovascular medication.

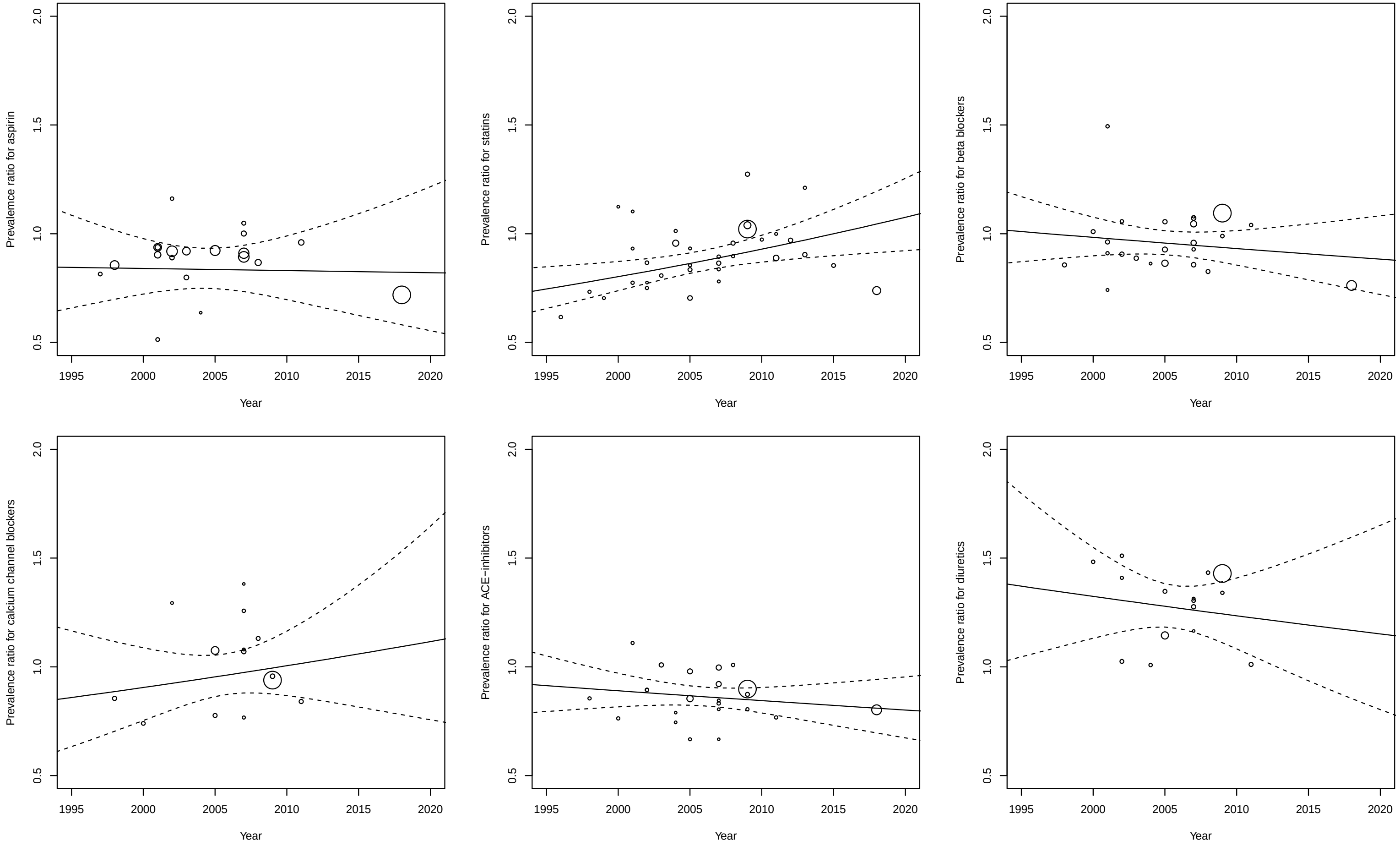

Bubbles are individual studies; diameters of the bubbles are proportional to studies weight for analysis.

Figure S5. Women-to-men prevalence ratio of cardiovascular medication prescription, stratified by CVD status.

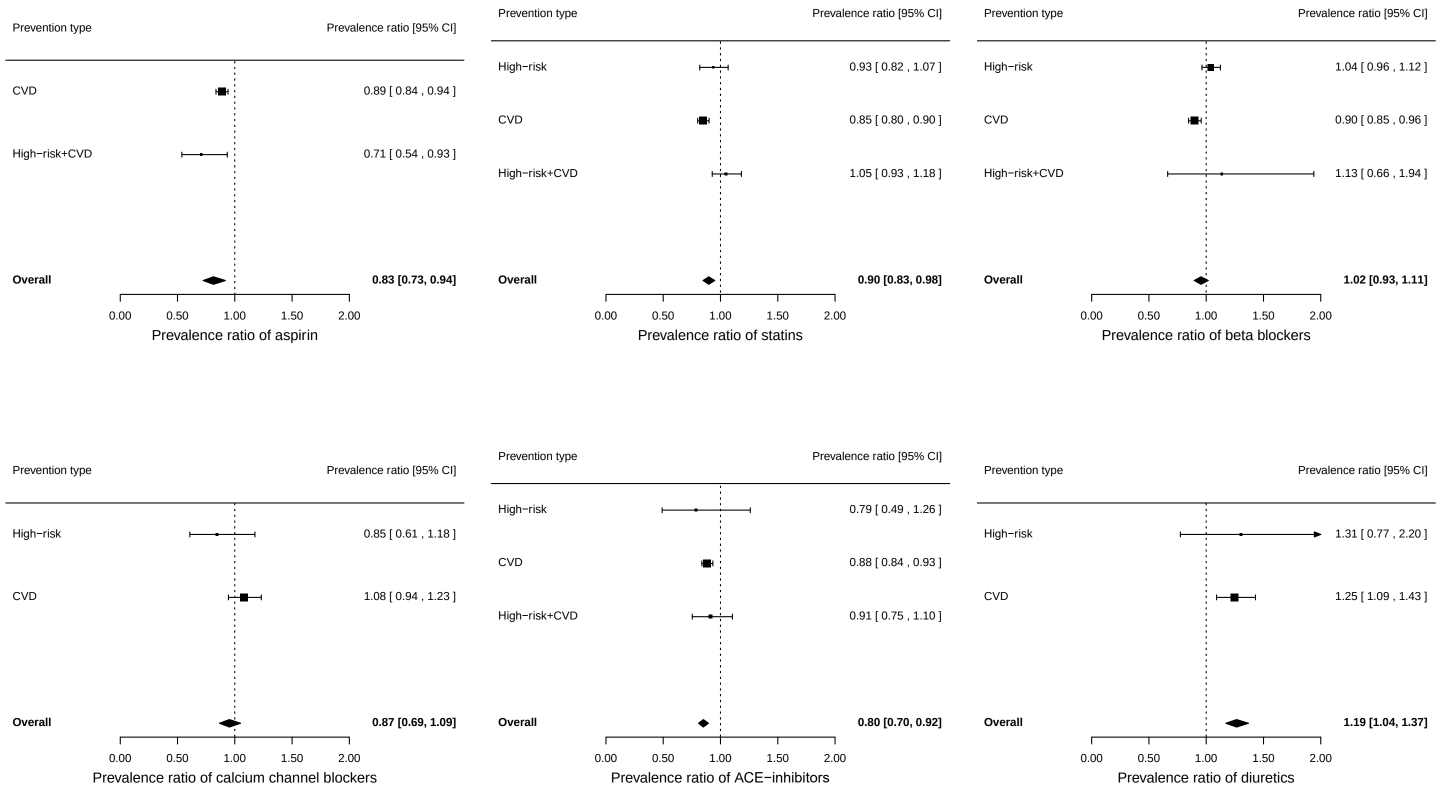

For each study, the square is centered on the women-to-men prevalence ratio and the horizontal lines show the associated 95% confidence interval. The diamond indicates the pooled summary and its 95% confidence interval.

Figure S6. Women-to-men prevalence ratio of aspirin prescription, stratified by CVD status.

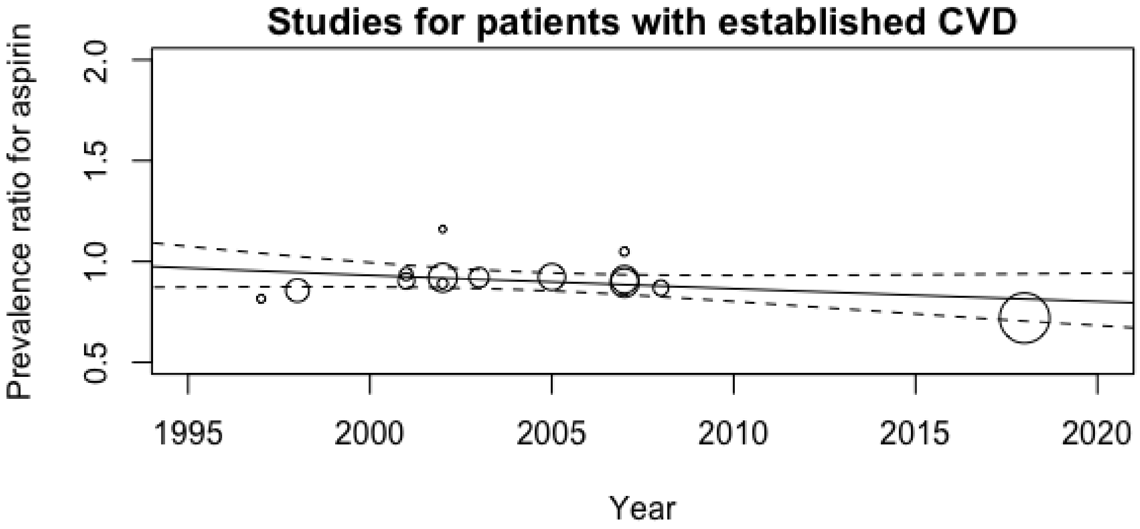

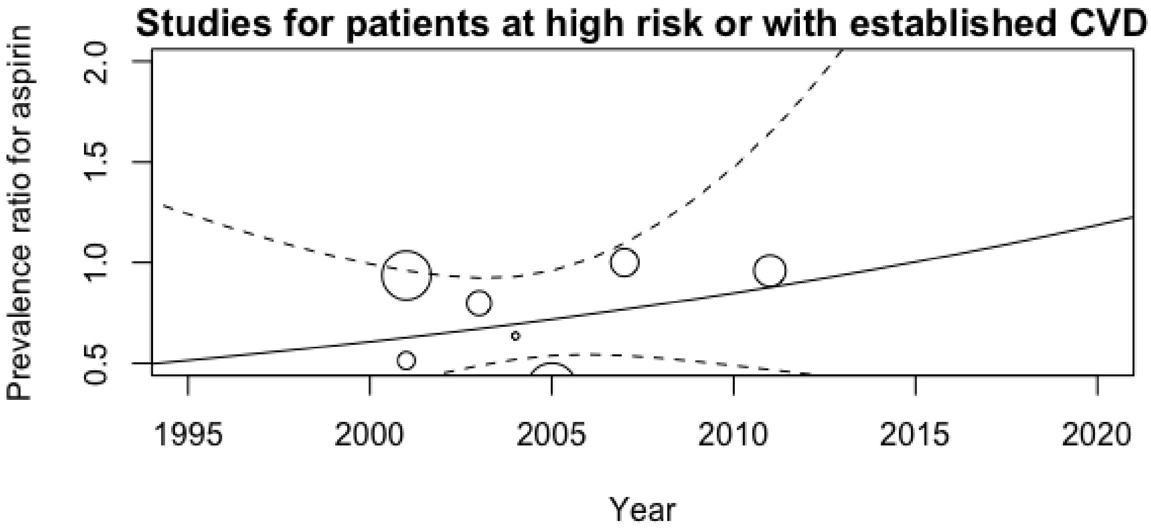

For each study, the square is centered on the women-to-men prevalence ratio and the horizontal lines show the associated 95% confidence interval. The diamond indicates the pooled summary and its 95% confidence interval.

Figure S7. Women-to-men prevalence ratio of statin prescription, stratified by CVD status.

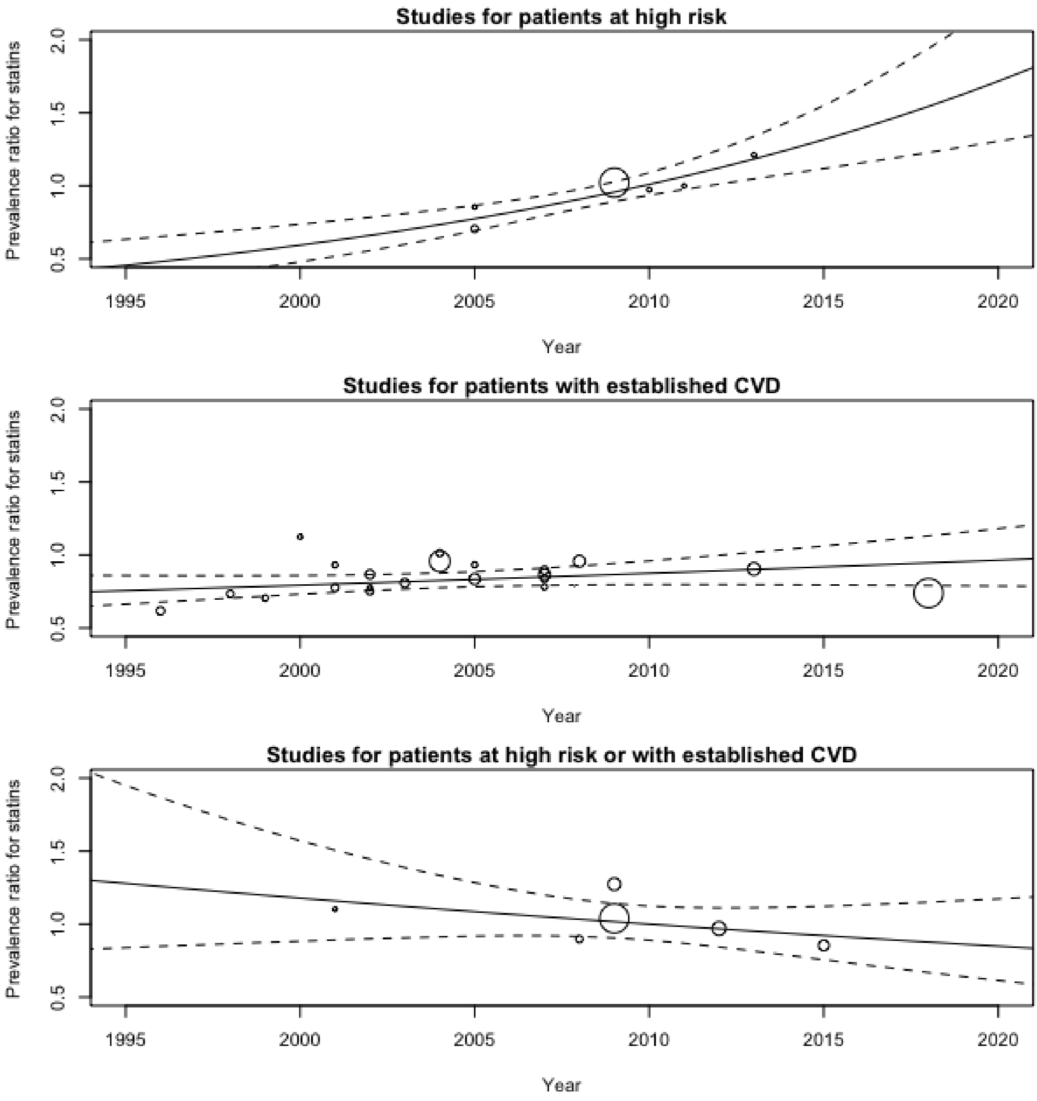

For each study, the square is centered on the women-to-men prevalence ratio and the horizontal lines show the associated 95% confidence interval. The diamond indicates the pooled summary and its 95% confidence interval.

Figure S8. Women-to-men prevalence ratio of beta blocker prescription, stratified by CVD status.

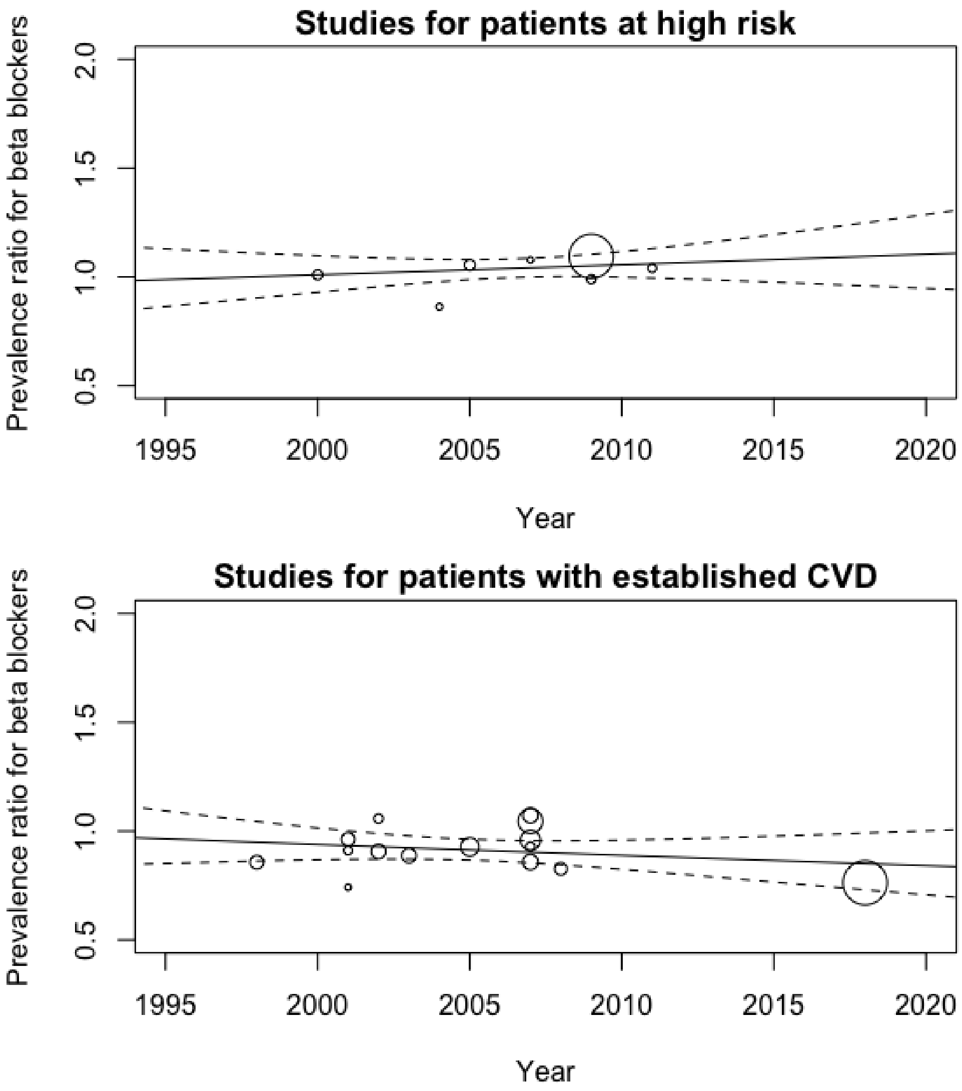

For each study, the square is centered on the women-to-men prevalence ratio and the horizontal lines show the associated 95% confidence interval. The diamond indicates the pooled summary and its 95% confidence interval.

Figure S9. Women-to-men prevalence ratio of calcium channel blocker prescription, stratified by CVD status.

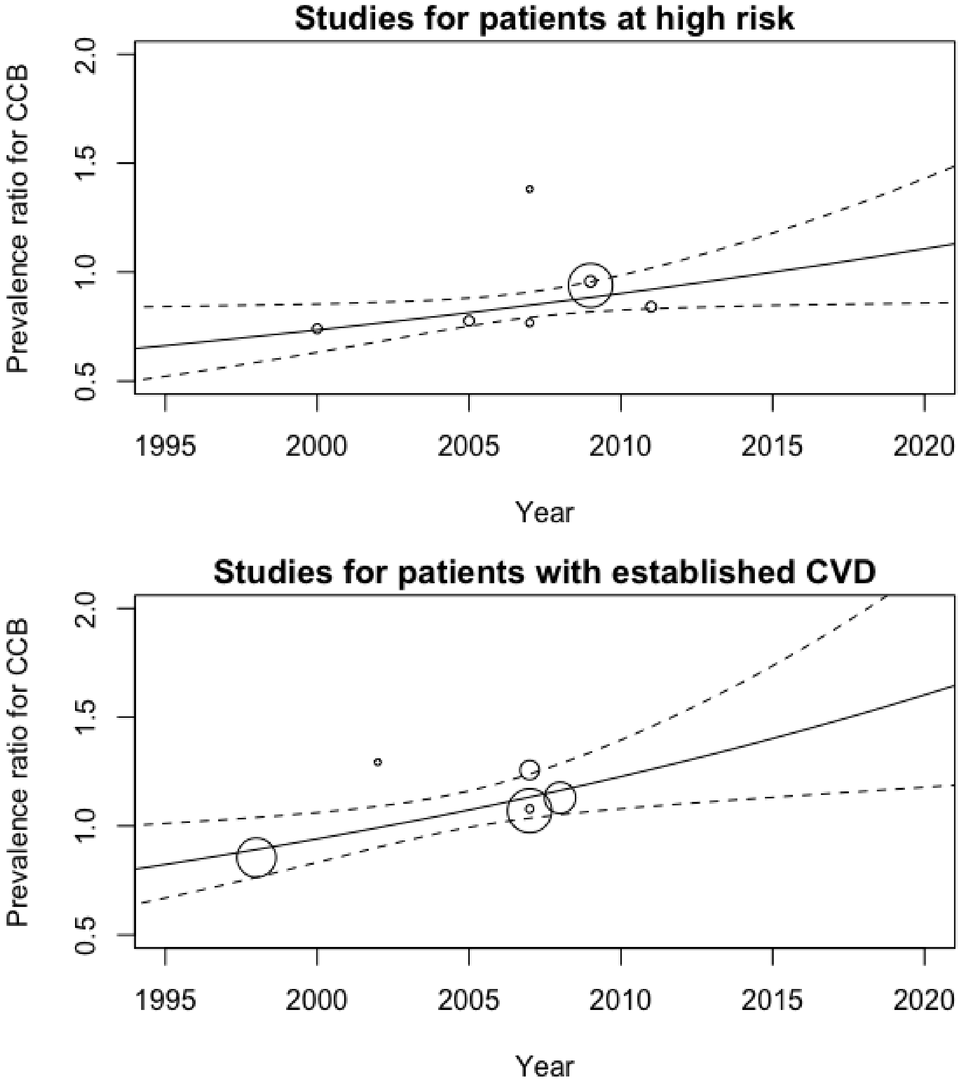

For each study, the square is centered on the women-to-men prevalence ratio and the horizontal lines show the associated 95% confidence interval. The diamond indicates the pooled summary and its 95% confidence interval.

Figure S10. Women-to-men prevalence ratio of ACE-inhibitor prescription, stratified by CVD status.

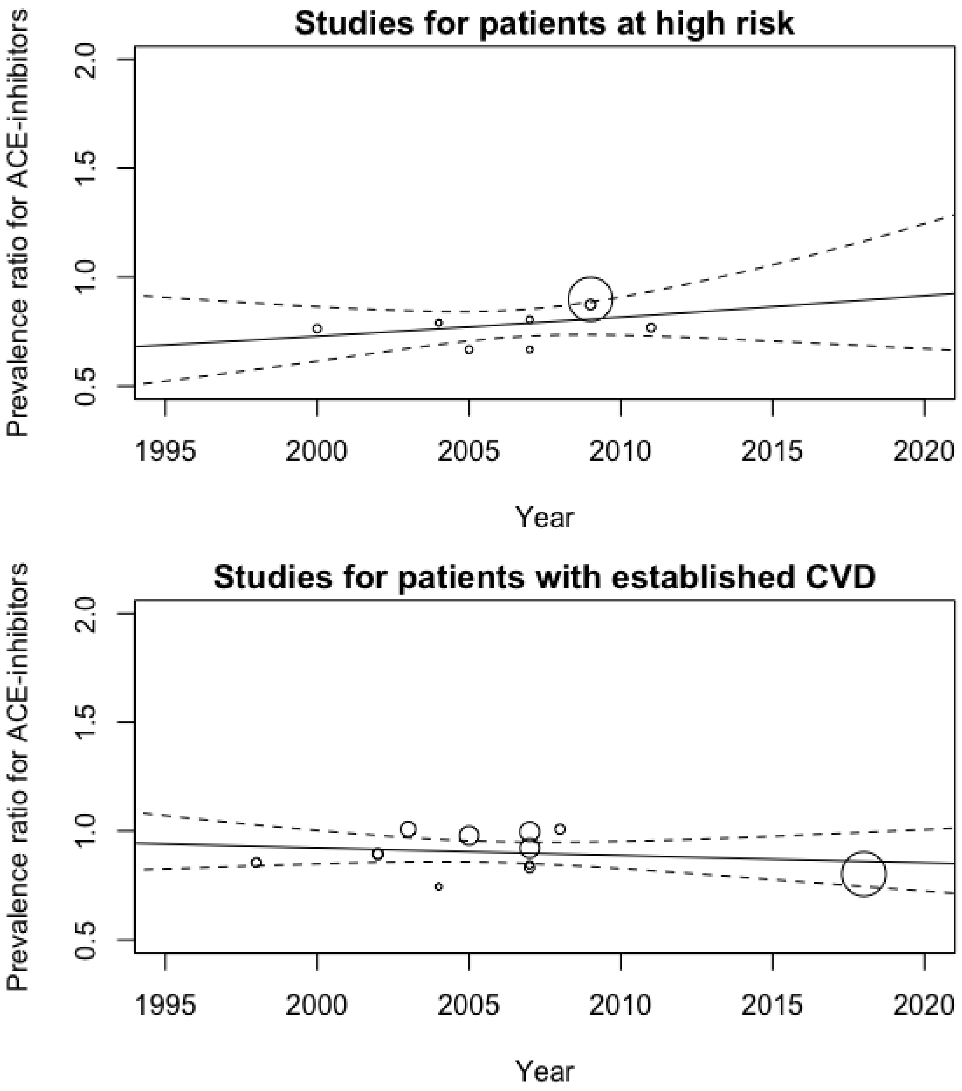

For each study, the square is centered on the women-to-men prevalence ratio and the horizontal lines show the associated 95% confidence interval. The diamond indicates the pooled summary and its 95% confidence interval.

Figure S11. Women-to-men prevalence ratio of diuretics prescription, stratified by CVD status.

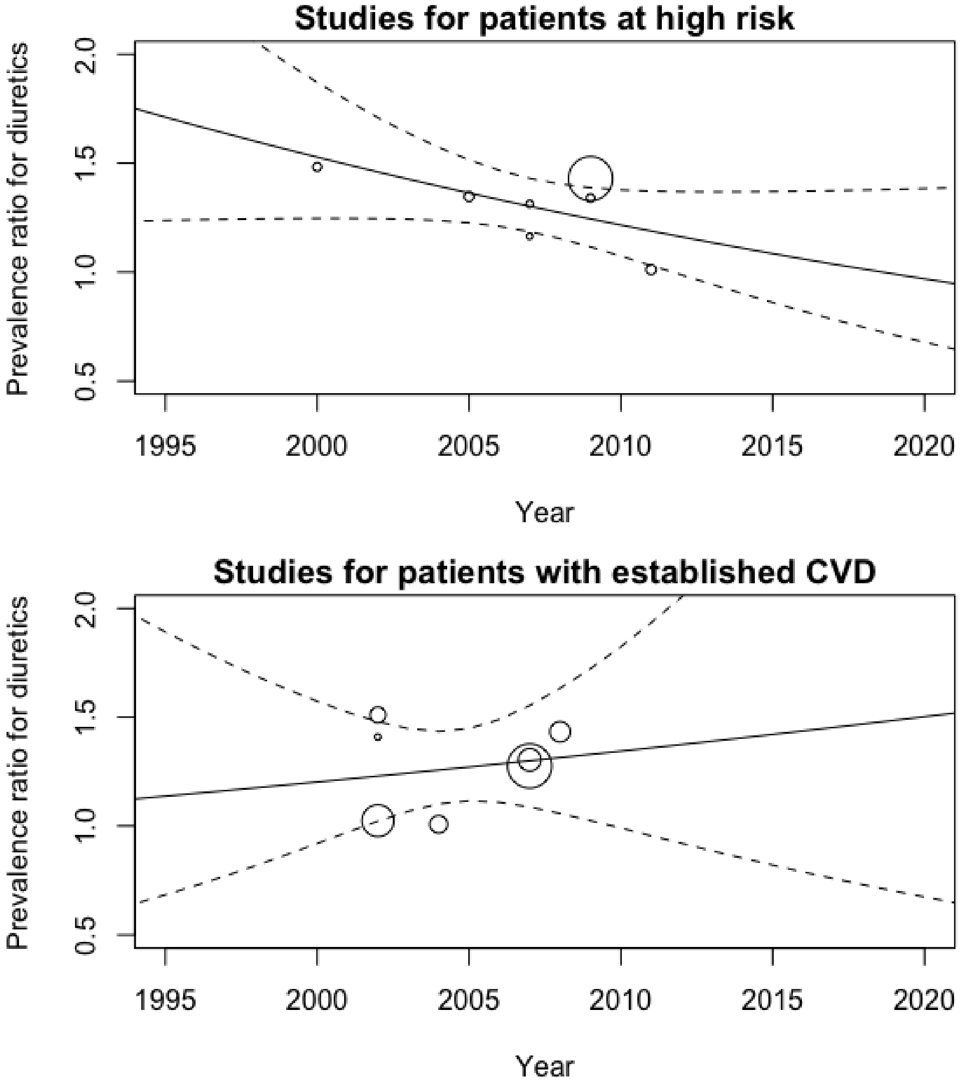

For each study, the square is centered on the women-to-men prevalence ratio and the horizontal lines show the associated 95% confidence interval. The diamond indicates the pooled summary and its 95% confidence interval.
